# Cross-Platform Evaluation Reveals Sample Preparation-Driven Biological Biases despite Advances in Mass Spectrometry Performance in Serum Proteomics

**DOI:** 10.1021/acs.jproteome.6c00493

**Published:** 2026-09-10

**Authors:** Drosos Katsavelis, Marieke G. C. van der Hart, Justina C. Wolters, Hjalmar P. Permentier, Peter L. Horvatovich, Thomas I.F.H. Cremers

**Affiliations:** † Quantall B.V., L.J. Zielstraweg 1, 9713 GX Groningen, The Netherlands; ‡ Department of Analytical Biochemistry, University of Groningen, Antonius Deusinglaan 1, 9713 AV Groningen, The Netherlands; § Department of Pediatrics, University of Groningen, University Medical Center Groningen, Antonius Deusinglaan 1, 9713 AV Groningen, The Netherlands

**Keywords:** serum proteomics, timsTOF HT, Orbitrap Exploris 480, quantitative performance, depth of proteome, reproducibility, biological pathways

## Abstract

Advances in mass spectrometry (MS) instrumentation and protein extraction technologies are reshaping proteomics; however, whether improved analytical performance alters the biological information accessible from different sample preparation strategies remains unclear. Here, we performed a cross-platform evaluation using identical benchmark serum samples previously analyzed on an Orbitrap Exploris 480 platform, including the newly introduced Seer Proteograph ONE workflow, to determine how MS advances and sample preparation influence analytical performance and biological interpretation. Across all preparation strategies, the timsTOF HT improved analytical performance by increasing protein and peptide identifications, reducing missing values, and improving linearity in spike-in experiments, particularly for low-abundance proteins. Despite these improvements, sample preparation remained the major determinant of the accessible serum proteome. Functional enrichment analysis revealed reproducible workflow-specific biases across MS platforms: nanoparticle-based enrichment expanded access to a broader range of serum proteins and improved detection of clinically relevant signaling-associated proteins, including cytokine- and chemokine-related marker classes, whereas the depletion-based strategy showed a distinct functional profile enriched for receptor-related activities. Together, these findings demonstrate that MS advances enhance analytical performance but do not eliminate sample preparation-driven differences in biological accessibility. This study provides a framework for selecting serum proteomics workflows according to specific biomarker and pathway objectives.

## Graphical abstract

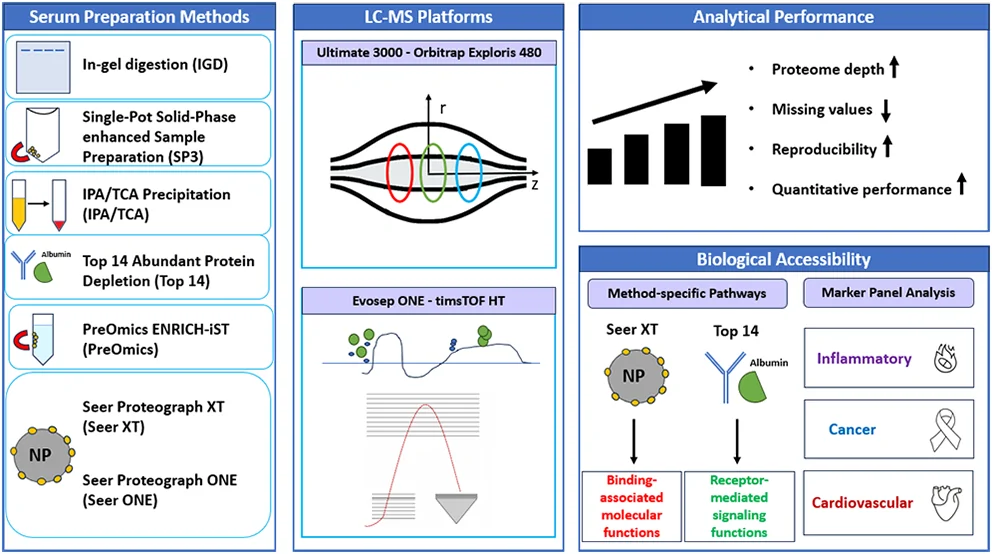

## Introduction

Liquid chromatography mass spectrometry (LC-MS/MS)–based proteomics has become a central analytical approach for the comprehensive characterization of complex biological systems, enabling large-scale protein identification and quantitative analysis across diverse biological matrices.^1–3^ Continuous advances in MS instrumentation, chromatography and data analysis have substantially increased analytical depth and throughput, positioning proteomics as a powerful tool for systems biology, translational research and access to biomarker-relevant proteins. Despite these advances, the analysis of human serum remains particularly challenging due to its vast dynamic range, which spans more than ten orders of magnitude in protein abundance, and the overwhelming presence of a limited number of highly abundant proteins that mask signals from lower-abundance species.^4–6^

To address these challenges, a wide range of serum preparation workflows has been developed. Conventional approaches, including in-gel digestion and Single-Pot Solid-Phase Enhanced Sample Preparation (SP3), primarily enable non-specific protein extraction.^7,8^ Other strategies aim to reduce sample complexity and expand detection of lower-abundance proteins, including immunoaffinity depletion, precipitation-based clean-up methods, and more recently, enrichment strategies using engineered beads or nanoparticles.^9–11^ Each of these workflows introduces distinct biases in peptide recovery, abundance distributions and reproducibility, which can profoundly influence downstream quantitative performance. Consequently, systematic benchmarking studies comparing blood preparation methods have played a crucial role in guiding experimental design and establishing best practices in quantitative serum proteomics.^12–15^

In our previous study, we conducted a comprehensive comparison of 6 commonly used serum preparation workflows using an Orbitrap Exploris 480 mass spectrometer, evaluating depth of proteome coverage, reproducibility and linearity using controlled spike-in experiments.^13^ That work demonstrated that nanoparticle-based enrichment using the Seer Proteograph XT workflow consistently outperformed other methods in terms of proteome depth and quantitative linearity. In contrast, conventional workflows exhibited moderate to poor linearity, particularly for low-abundance spiked-in proteins.

Since then, MS instrumentation has continued to evolve rapidly, with recent platforms offering significant improvements in sequencing speed, sensitivity, ion utilization, and effective dynamic range. Trapped ion mobility spectrometry (TIMS)–enabled time-of-flight instruments combine ion mobility separation with parallel accumulation–serial fragmentation (PASEF), allowing efficient sampling of complex peptide mixtures while maintaining high quantitative precision.^16^ The Bruker timsTOF HT represents a further evolution of this technology, enabling increased throughput and improved tolerance to highly complex peptide backgrounds.^17^ However, whether these improvements in analytical performance translate into changes in the biological information accessible from different sample preparation workflows remains unclear.

Importantly, many comparative evaluations of sample preparation workflows and MS platforms rely on independently generated datasets, often using samples of different origin and diverse analytical pipelines. Such study designs make it difficult to discriminate biological variability from true methodological or instrumental effects and may confound interpretation of cross-platform differences.^18^ While previous studies have demonstrated that sample preparation strongly influences serum and plasma proteome coverage, most comparisons have been performed using a single MS platform.^13–15^ Therefore, it remains unclear whether advances in MS instrumentation can overcome or alter sample preparation-driven biases in the biological composition of the detectable serum proteome. Direct analysis of identical biological material across different MS platforms and sample preparation strategies provides a unique opportunity to separate improvements in analytical performance from changes in biological accessibility.

In the present study, we revisit our previously established serum proteomics benchmark by reanalyzing the same spiked-in human serum samples on a Bruker timsTOF HT mass spectrometer, while retaining the original Orbitrap Exploris 480 data for direct comparison. In addition, we evaluate the performance of the newly introduced Seer Proteograph ONE workflow. By systematically comparing multiple sample preparation workflows across two fundamentally different MS platforms, we assess how improvements in MS acquisition influence proteome depth, reproducibility and response linearity across a certain concentration range. Beyond analytical performance, we investigate whether sample preparation-dependent differences in biological accessibility are preserved across MS technologies through global biological pathway enrichment and curated inflammatory, cancer and cardiovascular marker panels. Through this integrated analytical and functional comparison, we aim to establish how instrumentation and sample preparation contribute differently to serum proteomics performance and provide guidance for selecting workflows according to specific biomarker and pathway objectives.

## Experimental Section

### Reagents, Materials and Instrumentation

All reagents, consumables and instrumentation used for the sample preparation workflows have been previously described^13^ and the complete list is provided in Supporting Information. For the Seer ONE workflow, sample preparation was performed using the SP200 automation platform.

### Experimental Design

For the purpose of this study, 6 different serum protein extraction methods were compared between 2 mass spectrometry platforms, the Orbitrap Exploris 480 and the timsTOF HT for their depth of coverage, reproducibility and quantitative performance. Serum samples corresponding to these workflows were prepared in our previous study^13^ and were reused here without modification. Briefly, the following sample preparation methods were obtained: (a) Whole proteome analysis methods: In-gel digestion (IGD) and Single-Pot Solid-Phase-enhanced Sample Preparation (SP3), (b) depletion methods: Top 14 Abundant Protein Depletion (Top 14) and Isopropanol/Trichloroacetic Acid (IPA/TCA) precipitation and (c) enrichment methods: PreOmics ENRICH-iST (PreOmics), and Seer Proteograph XT (Seer XT). Three technical replicates (4 for Seer XT) were analyzed for 4 spike-in concentrations (Spikes 1-4) of 7 serum proteins, along with a non-spiked control, resulting in 15 samples (20 samples for Seer XT) per extraction method. The control group consisted of pooled human serum without added spike-in proteins. Spike-in proteins and concentration ranges are shown in Supporting Table S1. Furthermore, the newest Seer enrichment approach, Seer Proteograph ONE, was prepared and evaluated on the timsTOF HT platform, using 3 technical replicates per condition resulting in 15 samples in total.

### Serum Preparation

Spike-in serum samples used in this study were generated in the original publication as previously described.^13^ Briefly, the required amounts of purified proteins were combined to generate the highest concentration mixture (Spike 1), followed by serial 2-fold dilutions in PBS to produce Spikes 2–4. Each spike level was added to separate aliquots of 1.2 ml pooled serum at a 1:9 (v/v) ratio to achieve the final target concentrations. A control group was prepared by adding an equivalent volume of PBS to pooled serum to maintain consistent matrix composition across all conditions. Peptide aliquots designated for subsequent analysis on the timsTOF HT platform were stored at −80 °C and were not subjected to freeze–thaw cycles prior to LC-MS/MS measurements.

For evaluation of the Seer Proteograph ONE workflow, an independent spike-in serum batch was prepared from the same pooled serum source following the identical spike-in protocol and concentration scheme to ensure direct comparability. All prepared serum batches were aliquoted and stored at −80 °C until further processing.

### Sample Preparation Methods

The peptide samples from all sample preparation workflows (IGD, SP3, Top 14, IPA/TCA, PreOmics and Seer XT) were generated in the original publication^13^ and were reused here without modification for analysis on the timsTOF HT platform. The Seer ONE workflow was evaluated for the first time in this study and was applied exclusively to the timsTOF HT platform.

### Seer Proteograph ONE Workflow (Seer ONE)

Seer ONE workflow was performed based on the vendor’s instructions. Briefly, 120 μl of each serum sample was aliquoted into a well of a 96-well plate which was transferred to the SP200 instrument. Each sample was incubated with Proteograph ONE nanoparticles (NPs) for 1 h to allow for corona formation. Once the incubation ended, a magnetic field was used to accumulate the superparamagnetic NPs followed by a series of gentle washes to remove non-specific and weakly bound proteins as well as other contaminants. NP-bound proteins were reduced, alkylated and digested with Trypsin/Lys-C directly on the Proteograph ONE NPs to generate tryptic peptides for downstream LC-MS analysis. A second proprietary engineered particle solution aggregated the peptides to clean up and desalt the digestion mixture. The resulting peptides were eluted from the particles into a collection plate using an organic solvent/water mixture.

### Determination of Peptide Concentration

The peptide solutions that have been previously measured by Orbitrap Exploris 480 instrument, were determined as previously described^13^ following digestion and peptide cleanup. Briefly, the Pierce Quantitative Colorimetric Peptide Assay was used for all methods except Seer XT and all samples were adjusted to an estimated concentration of 300 ng/μL (150 ng/μL for Top 14). For Seer XT, peptide yield was determined by the Pierce Fluorescent Assay Kit (p/n 23290) and peptide solutions were diluted to a final concentration of 100 ng/μL.

For analysis on the timsTOF HT platform, peptide solutions derived from the original study were diluted to a uniform concentration of 25 ng/μl. Peptide yields from Seer ONE workflow were determined by the Pierce Fluorescent Assay Kit as in the case of Seer XT. The peptides were then dried down in a vacuum concentrator and reconstituted in 0.1% FA in Milli-Q to a final concentration of 25 ng/μl prior to LC-MS/MS analysis. Peptide concentration measurements were used to ensure consistent loading within each workflow.

### LC-MS/MS Instrumentation and Settings

LC–MS/MS data analyzed in this study were generated using 2 mass spectrometry platforms. Raw data acquired in a previous study on an Orbitrap Exploris 480 mass spectrometer were reused for comparative analysis, while all new measurements were performed exclusively on a timsTOF HT platform as described below.

### Orbitrap Exploris 480

Samples were analyzed on an Orbitrap Exploris 480 mass spectrometer (Thermo Fisher Scientific, USA) coupled to an Ultimate 3000 RSLCnano (Thermo Fisher Scientific, USA) as described previously.^13^ Briefly, 300 ng of peptide samples were separated using a 50 cm × 75 μm Acclaim C18 PepMap 100 nanocolumn packed with 3 μm particles (Thermo Fisher Scientific, Waltham, USA) with a 90 min gradient and analyzed in DIA mode.

#### TimsTOF HT

Samples were analyzed on a Bruker timsTOF HT mass spectrometer (Bruker, Germany) coupled to an Evosep One LC system (Evosep Biosystems, Denmark). Twenty μL of peptides (500 ng) were loaded on Evotips (Evosep Biosystems, Denmark) and separated on a PepSep C18 column of 15 cm × 150 μm, 1.5 μm (Bruker, Germany) in Bruker column toaster (40 °C) using a 30 SPD gradient. Data were acquired in dia-PASEF mode with the following parameters: MS1 scan range of 100-1700 m/z; MS2 scan range of 365–1325 m/z, 13 MS2 ramps, 39 MS2 windows without mass overlap, 1/K0 range of 0.70-1.30 V·s/cm^2^, ramp time and accumulation time of 100.0 ms, cycle time of 1.48 s and collision energy at 10.0 eV.

### Platform Comparison Standardization

Peptide loading amounts were optimized independently for each LC–MS/MS platform prior to data acquisition in order to operate each system within its optimal dynamic range and avoid detector saturation or underloading. Based on these optimization experiments, 300 ng of total peptide were injected for analyses performed on the Orbitrap Exploris 480 coupled to an Ultimate 3000 LC system, whereas 500 ng of total peptide were injected for analyses performed on the timsTOF HT coupled to an Evosep One LC system. These instrument-specific loading amounts were selected to maximize quantitative performance for each platform based on identification depth, reproducibility (measured by the coefficient of variation) and quantitative completeness. The selected loading amounts were then applied consistently across all sample preparation workflows analyzed on the respective platforms.

During analysis of the Seer XT dataset acquired on the timsTOF HT platform, a small number of samples exhibited aberrant chromatographic behavior (low signal and wide peak shape) and reduced proteome coverage, resulting in their exclusion from downstream analysis. Specifically, one replicate from Spike 4, two replicates from Spike 2, and two replicates from Spike 3 showed distorted or incomplete chromatograms and were removed prior to data analysis. These deviations were not observed in the corresponding Orbitrap dataset from the original study and are likely attributable to technical issues introduced during sample handling or LC–MS/MS acquisition. Exclusion of these samples improved data consistency and did not alter the overall trends observed for proteome coverage, reproducibility or quantitative linearity.

### Proteome Analysis

#### Protein Group Identification and Quantification

The identification of spectra from both instruments was performed with a human UniprotKB reviewed Swiss-Prot database (August 2025, 20.420 entries) by the software Spectronaut 20 (Biognosys AG, Zurich, Switzerland), version 20.4. The rest of the settings were selected as described previously.^13^ Briefly, methionine by oxidation and N-terminal acetylation were allowed as variable modifications. Carbamidomethylation on cysteine was selected as fixed modification for all methods except for IPA/TCA precipitation, in which reduction and alkylation were not performed. Only specific cleavage by trypsin was allowed with a maximum of two missed cleavages. For the PreOmics and Seer protocols, Lys C was selected in combination with trypsin. For Orbitrap Exploris 480 DIA data, label-free quantification was performed at the MS1 level, as the DIA method and acquisition parameters were specifically optimized for MS1-based quantification, in line with previous work demonstrating improved quantitative performance under these conditions.^19^ For timsTOF HT data, label-free quantification was performed using the default MS2-based quantification strategy in Spectronaut, which is the recommended and widely adopted approach for PASEF-based DIA data on timsTOF instruments. Normalization across runs was obtained using the software’s default local normalization strategy.

#### Data Filtering

Normalized protein group intensities were imported to Perseus (version 2.1.5.0)^20^ in order to filter for missing values. Only protein groups detected in at least 60% of all the replicates per sample preparation method were selected for downstream analysis.

#### Quantitative Performance of Spiked-in Proteins

Quantitative performance was evaluated through linear regression analysis for each spiked-in protein across the different concentrations and sample preparation methods. The spiked proteins were grouped into three concentration ranges based on their expected abundance in serum: (i) High concentration proteins (0.5–4.0 ng/μL; CRP and S100A8), (ii) medium concentration proteins (0.25–2.0 ng/μL; LYZ and PPIA), and (iii) low concentration proteins (0.05–0.4 ng/μL for GLO1 and PRKAR1A, and 0.0625–0.5 ng/μL for YWHAE).

For each spiked-in protein and workflow, regression analyses were performed using all individual replicate measurements across spike levels. Replicates failing predefined quality criteria (CV > 40% and missing values >60% across technical replicates per spiked concentration) were considered outliers and excluded prior to regression analysis. The same criteria were applied to all workflows and instruments. In cases where endogenous signal was detected in control samples, the endogenous concentration was estimated using the standard addition method by dividing the y-intercept of the regression line by the slope.^21^

### Statistical Analysis

The upset plots were generated by the R package “UpSetR”. Protein groups were subjected to a gene set enrichment analysis (GSEA) performed by the web tool g:Profiler.^22^ GO term enrichment was performed with g:SCS algorithm which performs multiple testing correction for *p*-values gained from GO and pathway enrichment analysis and corresponds to an experiment-wide threshold of *a* = 0.05. Linear regression analysis was performed with RStudio, version 2026.01.0.

## Results and Discussion

### MS Instrumentation Significantly Affects Analytical Performance

#### Protein Group and Peptide Identifications

Proteome coverage was evaluated at both the protein group and peptide level after filtering for missing values for each sample preparation method, thereby retaining only consistently quantified identifications across replicates. Across all workflows common to both platforms, the timsTOF HT consistently yielded higher numbers of identified protein groups and peptides compared to the Orbitrap Exploris 480 (Figure 1A). Among non-affinity and non-enrichment–based workflows, modest protein group coverage was observed for IGD, SP3, and IPA/TCA, with protein group identifications ranging from 652 to 755 on the timsTOF HT and from 352 to 429 on the Orbitrap 480 platform. Affinity- and enrichment-based strategies resulted in substantially increased coverage, with Top 14 depletion and PreOmics workflows yielding up to 1400 and 1226 protein groups on the timsTOF HT, respectively, compared to 471 and 709 protein groups on the Orbitrap Exploris 480. Consistent with previous findings,^13^ nanoparticle-based enrichment using the Seer XT workflow achieved the highest depth of coverage on both platforms, identifying 3,009 protein groups on the timsTOF HT and 1981 protein groups on the Orbitrap Exploris 480. A similar trend was observed at the peptide level. Across workflows, peptide identifications were consistently higher on the timsTOF HT, ranging from 7095 to 25,569 peptides, compared to 3657 to 17,325 peptides on the Orbitrap. The Seer ONE workflow, evaluated exclusively on the timsTOF HT, achieved coverage comparable to Seer XT, identifying 2475 protein groups and 24,101 peptides, further highlighting the high proteome depth attainable using nanoparticle-based enrichment in combination with high-speed PASEF acquisition. The full lists of protein group and precursor identifications for all sample preparation methods on both MS platforms can be found in Files S1 and S2.

**Figure 1:**
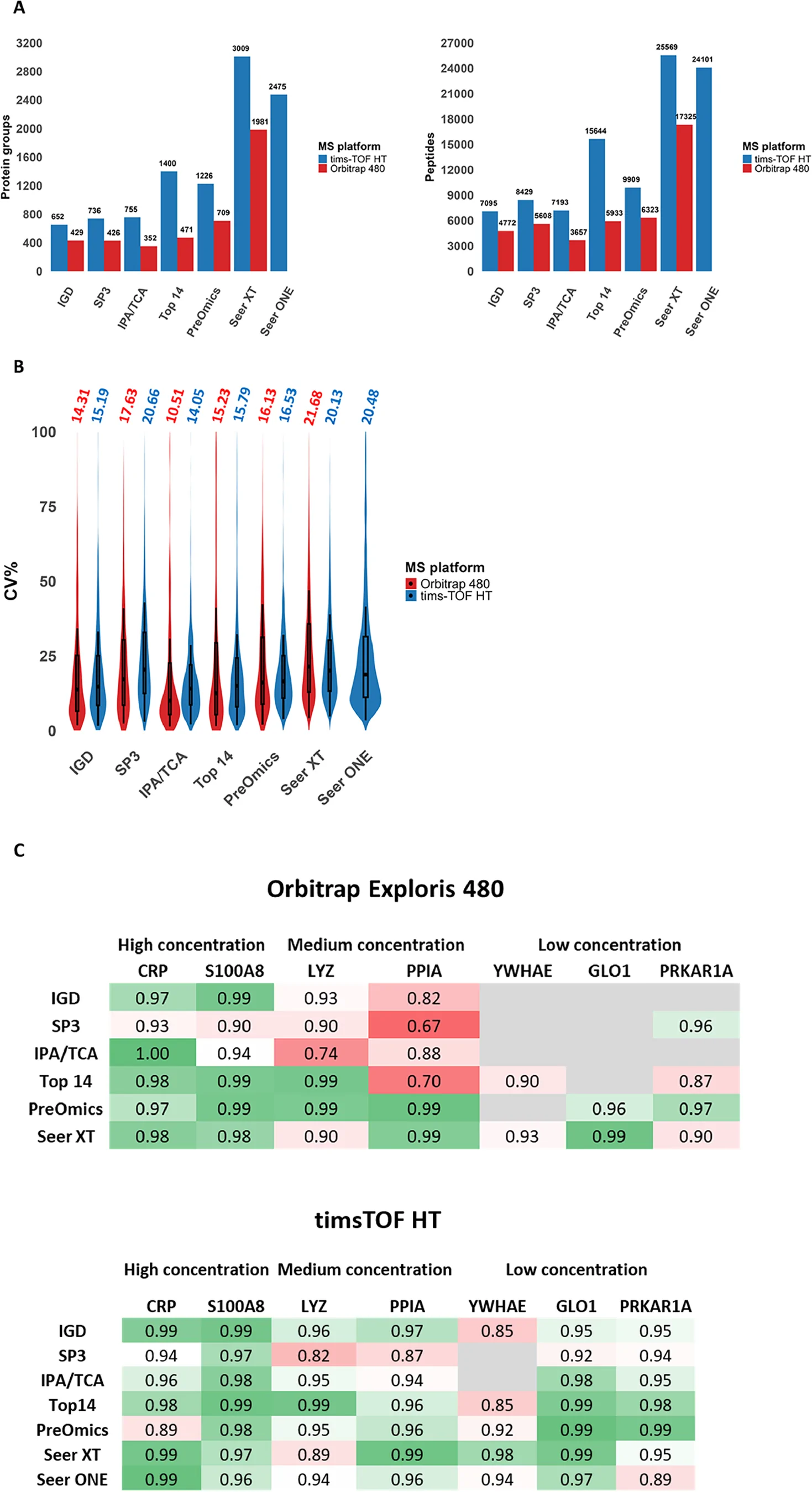
(A) Protein group and peptide identification across all serum sample preparation methods on timsTOF HT and Orbitrap Exploris 480. (B). Reproducibility of serum sample preparation methods across timsTOF HT and Orbitrap Exploris 480 as accessed by the coefficient of variation (CV%) for protein group intensities. Median values are indicated on top while medians and interquartile ranges are shown in the form of a box plot. (C) Heatmap representation of coefficients of determination (*R* ^2^) obtained from linear regression analysis of spiked-in protein concentrations across all serum preparation workflows and MS platforms (timsTOF HT and Orbitrap Exploris 480). Green color indicates high linearity, white color indicate modest linearity and red color shows poor linearity. Grey cells indicate conditions where *R* ^2^ values could not be calculated due to missing measurements or insufficient data points.

To further compare quantitative sampling between platforms, the average number of peptides identified per protein group for each workflow was extracted from Spectronaut analysis (Supporting Table S2). Despite the higher proteome depth achieved by the timsTOF HT, the average peptide coverage per protein group was highly comparable between the two platforms across all workflows, indicating that the increased number of protein identifications was not accompanied by a systematic increase in peptide evidence per protein. Instead, the improved analytical performance primarily reflected expansion of the detectable proteome while maintaining similar peptide support for protein identification. Across both platforms, nanoparticle-based enrichment with Seer XT yielded the lowest average peptide coverage per protein group. This is consistent with its substantially greater proteome depth, as expansion into the low-abundance region of the serum proteome is expected to increase the proportion of proteins supported by fewer confidently detected peptides.

Precursor features uniquely identified on each LC-MS platform were compared across all sample preparation workflows (File S3). As expected, the timsTOF HT consistently detected substantially more platform-specific precursor features than the Orbitrap Exploris 480 across all workflows. Despite this difference in precursor numbers, the distributions of precursor m/z, precursor mass and retention time were highly comparable between platforms and across sample preparation methods, with no consistent physicochemical bias observed. In contrast, precursor charge state distributions showed a reproducible difference, with the Orbitrap Exploris 480 identifying a greater proportion of singly charged precursor ions across all workflows, whereas the timsTOF HT predominantly detected multiply charged precursor species. A possible explanation is that the trapped ion mobility separation (TIMS) implemented in the timsTOF HT preferentially transmits and identifies multiply charged peptide precursors while reducing the contribution of singly charged ions. Interestingly, a similar effect has been reported for Orbitrap-based workflows employing high-field asymmetric waveform ion mobility spectrometry (FAIMS), although FAIMS was not used in the present study, suggesting that gas-phase ion mobility separation may contribute to the observed differences in precursor charge-state distributions.

The median peptide yields per serum input for all sample preparation workflows, including Seer Proteograph ONE, are provided in Supporting Table S3. Similar to the previously evaluated Seer XT workflow, Seer ONE nanoparticle-based enrichment produced relatively low peptide yields compared to conventional extraction methods while achieving substantially greater proteome depth, indicating that the loss of material was primarily due to the loss of high-abundance proteins during the enrichment process.

The proportion of missing values was assessed prior to missing-value filtering to evaluate data completeness across sample preparation workflows and LC-MS/MS platforms (Supporting Figure S1). Across workflows common to both platforms, missing values were generally lower on the timsTOF HT compared to the Orbitrap platform, particularly for IGD and Seer XT. The higher proportion of missing values observed for the Seer ONE workflow (11.03%) compared to Seer XT (2.13%) on the timsTOF platform, despite the similar number of peptide identifications, likely reflects enrichment methodological differences between the 2 workflows. Seer ONE employs a modified enrichment strategy, including a single nanoparticle solution and a shortened proteolytic digestion protocol, which may alter peptide recovery, abundance distributions and feature stability across replicates. Importantly, subsequent filtering reduced missing values to below 4% for all workflows in both instruments, enabling robust downstream quantitative and statistical (e.g., linearity of spiked-in proteins) analyses.

Both platforms provided sufficient sampling density across all sample preparation strategies, with median values ranging from 7–10 MS1 points per peak for the Orbitrap Exploris 480 and 5–6 MS2 points per peak for the timsTOF HT. Because these values derive from different acquisition strategies (MS1-DIA versus dia-PASEF) and are therefore not directly equivalent, they were interpreted as indicators of adequate chromatographic sampling rather than as a direct comparison of quantitative performance.

#### Reproducibility

The reproducibility of each method on both instruments was assessed by the median coefficient of variation (CV%) for the protein group intensities across all replicates after missing-value filtering (Figure 1B). Across all workflows, median CVs ranged from 14.05% to 20.66% on the timsTOF HT and from 10.51% to 21.68% on the Orbitrap Exploris 480. Overall, reproducibility was comparable between platforms, with no systematic shift toward higher or lower median CVs for either instrument. Workflows such as IGD, Top 14, PreOmics and Seer XT exhibited similar median CVs on both platforms while IPA/TCA and SP3 displayed marginally higher median CVs on the timsTOF HT, although differences were small. These results indicate that quantitative reproducibility is primarily governed by the sample preparation workflow, while modern LC-MS/MS platforms provide broadly comparable precision when operated under optimized conditions.

To further dissect the contribution of LC-MS/MS platform to quantitative reproducibility, protein group-level CVs for each sample preparation method were evaluated separately for protein groups shared between the Orbitrap Exploris 480 and timsTOF HT and for protein groups uniquely identified on each platform (Supporting Figure S3). For protein groups commonly identified on both MS platforms, median CVs were consistently lower and the IQR was also narrower on the timsTOF HT compared to the Orbitrap Exploris 480 across all workflows. Protein groups uniquely identified on each platform exhibited substantially higher median CVs, consistent with their generally lower abundance and proximity to the detection limit and this effect was more pronounced for Orbitrap-specific protein groups. As expected, precursor-level median CVs were consistently higher than protein-level median CVs, reflecting the greater variability of individual precursor measurements compared with protein-level aggregated quantification (Supporting Table S11). Nevertheless, the relative performance of the evaluated workflows and platforms remained consistent with the protein-level analysis, and precursor-level variability did not alter the overall conclusions regarding quantitative reproducibility.

Although direct comparisons are limited because most benchmark studies have focused on plasma rather than serum, the protein identification numbers and quantitative reproducibility observed in this study are broadly consistent with previous reports using comparable LC-MS/MS workflows.^23–26^ Serum generally yields fewer protein identifications than plasma, likely reflecting differences in sample composition and processing, and our results fall within the expected range reported for contemporary serum and high-quality plasma proteomics datasets.^27,28^

A limitation of this cross-platform comparison is that the Orbitrap Exploris 480 and timsTOF HT datasets were generated using different LC-MS workflows, including differences in chromatographic configuration. However, previous multicenter studies have demonstrated comparable serum and plasma proteomics performance across different state-of-the-art LC systems, including Evosep One and Ultimate 3000 configurations, when coupled with high-resolution MS platforms, including Orbitrap Exploris 480. These studies reported similar proteome depth as well as robust quantitative reproducibility and data completeness across workflows.^29^ In addition, the peak capacity values (the number of chromatographic peaks that can theoretically be separated during a run) obtained across the different sample preparation workflows in this study were highly comparable between the Orbitrap Exploris 480 and timsTOF HT datasets for most of the sample preparation methods tested, suggesting that differences in chromatographic separation were unlikely to be the primary driver of the observed performance differences (Supporting Table S4). Therefore, while the individual contributions of LC configuration, acquisition strategy and MS platform cannot be completely separated in this study, the magnitude of the improvements observed with the timsTOF HT workflow is consistent with enhanced MS-based analytical capabilities.

#### Quantitative Performance Assessed Using Spiked-in Proteins

Overall, linear regression analysis of proteins spiked-in at various concentration levels demonstrated strong concentration-dependent responses across the evaluated workflow, with clear platform- and method- dependent differences in response linearity (Figure 1C and Supporting Figure S4). For high-concentration proteins (CRP and S100A8), both platforms achieved high linearity across most workflows, with *R* ^2^ values typically exceeding 0.95, indicating consistent quantitative response over the tested concentration range irrespective of sample preparation method or MS platform. Quantitative reproducibility was likewise high, with CVs generally below 15% across technical replicates (Supporting Tables S5–S6).

Differences between MS platforms became more pronounced at medium concentration ranges (LYZ and PPIA). On the timsTOF HT platform, linearity remained high across nearly all workflows, with *R* ^2^ values frequently exceeding 0.94. In contrast, Orbitrap Exploris 480 analyses showed greater variability across methods, particularly for IGD, SP3 and IPA/TCA, where one or more medium-abundance proteins exhibited reduced linearity. This reduced performance was also reflected by higher CV values for several workflow–protein combinations.

The largest platform-dependent differences were observed for the low-concentration proteins (YWHAE, GLO1 and PRKAR1A). On the Orbitrap Exploris 480 platform, detection of these proteins was highly method-dependent and often incomplete, preventing reliable regression analysis for several method–protein combinations. In contrast, the increased analytical sensitivity of the timsTOF HT enabled consistent detection and quantification of these low-abundance proteins for IGD, SP3 and IPA/TCA, allowing concentration-response relationships to be established and generally resulting in high response linearity together with acceptable quantitative reproducibility.

Among all evaluated workflows, Seer XT demonstrated the most consistent quantitative performance across both MS platforms. All 7 spiked proteins were detected across the tested concentration range, with *R* ^2^ values exceeding 0.95 for the majority of protein–platform combinations and generally low technical variability. These findings indicate that Seer XT maintained robust quantitative behavior while extending proteome coverage toward lower-abundance proteins.

For the high- and medium-concentration spike-in proteins, both platforms identified a similar number of peptides across most sample preparation workflows (Supporting Tables S9–S10). In contrast, differences became apparent for the low-abundance proteins, where the timsTOF HT consistently identified additional peptide sequences, particularly for IGD, SP3 and IPA/TCA, in which one or more low-abundance proteins were not detected on the Orbitrap Exploris 480. Conversely, workflows providing efficient low-abundance protein enrichment, including Seer XT, Seer ONE and Top14 depletion, showed comparable peptide support for the low-abundance spike-ins on both platforms. These observations are consistent with the improved sensitivity of the timsTOF HT for low-abundance proteins while confirming that the increased proteome depth was not associated with a systematic increase in peptide coverage for proteins that were already identified.

To further investigate platform-specific quantitative behavior, average intensity ratios (Orbitrap Exploris 480/timsTOF HT) were calculated across all spiked concentrations for the Seer XT workflow, which exhibited consistently high linearity on both instruments. Despite differences in absolute signal intensity, both platforms preserved similar relative quantitative trends across concentration levels for all spiked proteins except S100A8, which exhibited progressively higher Orbitrap/timsTOF HT intensity ratios with increasing concentration, indicating a stronger signal response on the Orbitrap Exploris 480 at higher abundance levels (File S4).

### Sample Preparation Drives Biological Accessibility of the Serum Proteome

#### Global Enrichment Analysis on Unique Protein Groups

To determine how sample preparation strategies influence downstream biological interpretation, Gene Ontology (GO) enrichment analysis was performed on unique protein group identifications per method and per instrument for the term Molecular Function. To minimize enrichment bias arising from comparison with the complete human proteome, the background universe was defined as the union of all protein groups identified across all sample preparation workflows and both MS platforms (*n* = 3875 annotated proteins). Only significantly enriched GO terms passing statistical correction were retained for interpretation. In addition, GO terms with term size greater than 1000 proteins were excluded to avoid overly general biological categories. Enriched pathways were ranked based on gene ratio (proteins involved in the specific pathway/all proteins used as input), and statistical significance was indicated using adjusted *p*-values. For direct comparison between instruments, downstream biological analyses focused on sample preparation workflows were evaluated on both the Orbitrap Exploris 480 and timsTOF HT platforms.

As shown in Figure 2A, IGD, SP3 and IPA/TCA produced fewer than 40 unique protein groups on both instruments and did not yield significant molecular function enrichment results after statistical correction. Similarly, PreOmics produced no significant enrichment despite identifying 90 and 62 unique proteins on the timsTOF HT and Orbitrap Exploris 480, respectively. Seer XT yielded the highest number of unique protein group identifications on both platforms (timsTOF HT: 1683; Orbitrap Exploris 480:1319). Enrichment analysis revealed a consistent functional landscape detected by both instruments (Supporting Figure S5).

**Figure 2:**
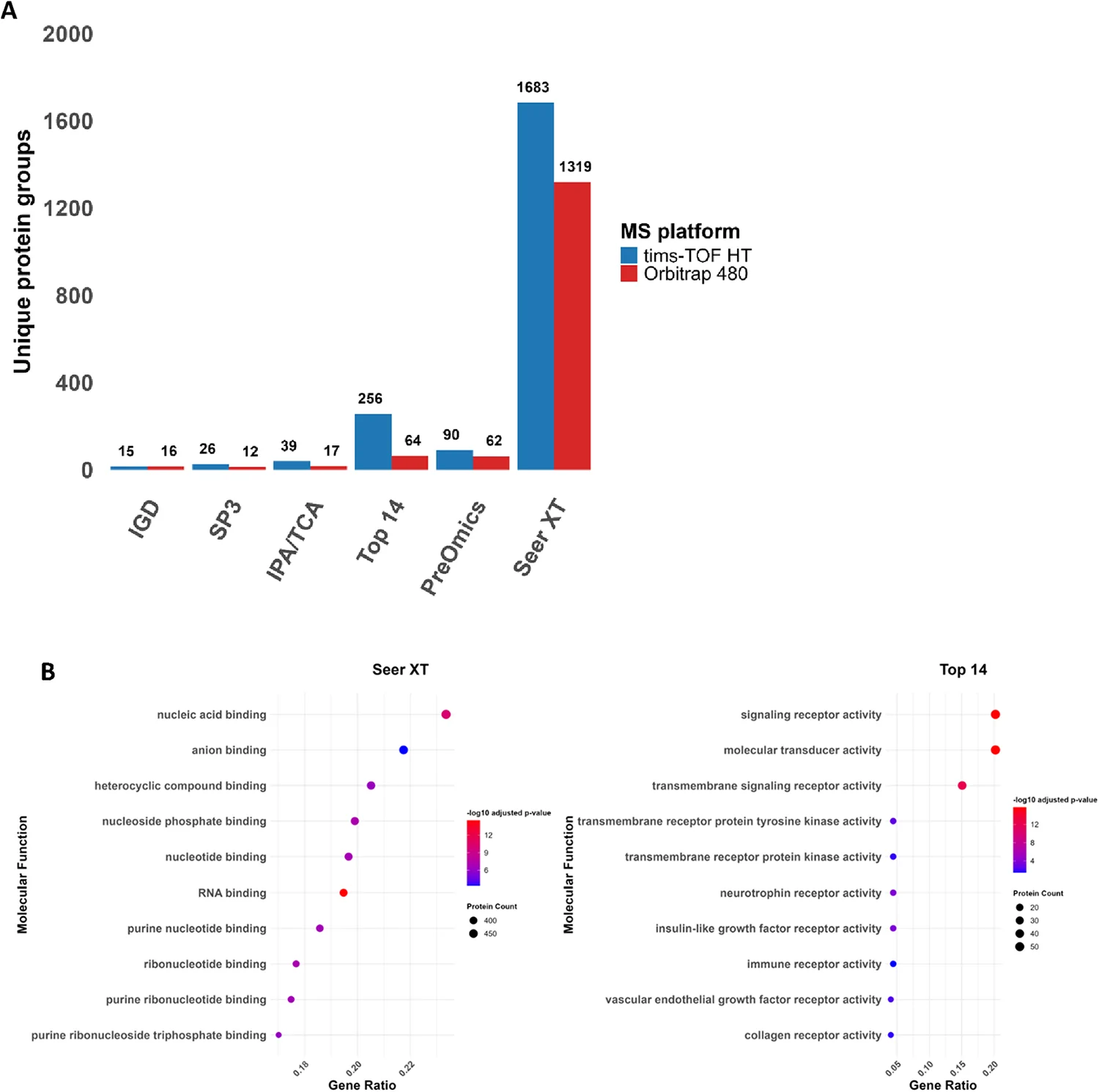
(A) Number of unique protein groups across all serum sample preparation methods on timsTOF HT and Orbitrap Exploris 480 MS platforms. (B) Top 10 enriched GO Molecular Function terms derived from unique protein groups identified by the Seer XT and Top 14 workflows. For each workflow, protein groups detected across both MS platforms were combined and duplicates removed prior to enrichment analysis.

To further assess robustness, unique protein groups identified by Seer XT on both instruments were combined (*n* = 2116 annotated proteins). The combined analysis reinforced and strengthened the same molecular functions (Figure 2B). Nucleic acid-, nucleotide- and RNA-binding activities emerged as the most significantly enriched term, indicating broad expansion of proteins involved in regulatory and binding-associated molecular functions. Top 14 depletion yielded fewer unique protein groups (timsTOF HT: 256; Orbitrap Exploris 480:64) but produced a coherent and biologically distinct enrichment profile. As in the case of Seer XT, proteins identified by both instruments enriched common molecular functions (Supporting Figure S5) and when the unique proteins from both platforms were combined (*n* = 272 annotated proteins), molecular transducer and signaling receptor activities, including immune receptor, receptor tyrosine kinase, collagen receptor and vascular endothelial growth factor receptor activities were among the significant enrichment processes with the highest gene ratios, highlighting a preferential representation of receptor-mediated signaling functions. The high concordance between platform-specific (Supporting Figure S5) and combined analyses (Figure 2B) indicates that the expanded protein coverage of the identified GOs observed with Seer XT and Top 14 depletion is driven primarily by the sample preparation strategy rather than instrument-dependent detection bias.

These differences likely arise from the fundamentally distinct enrichment principles of the two workflows. The nanoparticle-based Seer XT platform selectively captures proteins based on physicochemical interactions, enabling enhanced recovery of low-abundance proteins, while immunodepletion of the Top14 most abundant plasma proteins primarily reduces dynamic range by removing the 14 most abundant serum proteins while retaining a proteome biased toward moderate-to-high abundance proteins not targeted by the combined antibodies.

In order to evaluate differences between nanoparticle-based enrichment strategies, protein group identifications obtained with Seer XT and Seer ONE were compared on the timsTOF HT platform. A substantial overlap was observed (2008 protein groups), indicating that both workflows capture a common core serum proteome. However, Seer XT identified a larger number of unique proteins (*n* = 1,001) compared to Seer ONE (*n* = 467), suggesting increased depth of coverage. These differences likely reflect distinct protein corona compositions arising from nanoparticle surface chemistries, resulting in preferential capture of distinct protein subsets. In addition, Seer ONE is designed as a simplified, higher-throughput enrichment strategy rather than a direct replacement for Seer XT optimized for maximum proteome depth. The use of a single nanoparticle formulation together with a shorter digestion protocol may influence protein recovery and abundance distributions. We also acknowledge that the Seer ONE workflow was evaluated using an independently prepared serum batch generated according to the identical spike-in protocol rather than the original benchmark aliquots. Although every effort was made to standardize sample preparation and storage conditions, we cannot exclude the possibility that minor batch-to-batch variation contributed to the observed differences in proteome depth.

Interestingly, functional enrichment analysis of both Seer XT- and Seer ONE-specific protein groups using the experimentally derived background did not identify any significantly enriched molecular functions after multiple-testing correction. Despite the great number of unique protein groups identified by both Seer platforms, the absence of significant enrichment for either workflow suggests that the workflow-specific proteins are broadly distributed across diverse molecular functions rather than representing preferential enrichment of specific functional classes. Together with the substantial overlap between the 2 Seer workflows, these findings indicate that Seer XT and Seer ONE access a largely similar functional landscape of the serum proteome, with the primary difference being analytical depth rather than functional selectivity.

Potential MS instrument-specific effects were assessed through molecular function enrichment analysis on protein groups uniquely identified by the timsTOF HT platform for each sample preparation method, as shown by the protein group overlap analysis. Following background correction, no significantly enriched molecular functions were identified for IGD and PreOmics, whereas SP3 and IPA/TCA showed a limited number of significantly enriched receptor- and signaling-related functions. Top14 exhibited the broadest enrichment profile, while Seer XT showed enrichment for a single molecular function. Importantly, the receptor- and signaling-related functions identified for SP3 and IPA/TCA were largely recapitulated within the broader enrichment profile observed for Top14, indicating that the additional protein groups identified by the timsTOF HT primarily expanded proteome coverage within biological spaces already accessible through the corresponding sample preparation strategies rather than introducing fundamentally distinct functional signatures. Detailed results are provided in File S5.

#### Functional Characterization of Disease-Marker Proteins across Sample Preparation Strategies

To evaluate the capacity of each sample preparation strategy to recover clinically relevant proteins, curated marker panels representing inflammatory, cancer and cardiovascular biology were interrogated against the total proteome identified by each method and instrument. The inflammatory marker list (*n* = 557) was generated by querying the term “inflammatory response” in the PANTHER Classification System.^30^ Cancer-associated proteins (*n* = 213) and cardiovascular-associated proteins (*n* = 166) were extracted from the Human Blood Protein Atlas.^31^ The cancer panel comprised proteins linked to breast, colorectal, cervical, ovarian, prostate, lung and pancreatic cancers, while the cardiovascular panel included proteins associated with acute coronary syndrome, acute venous thromboembolism, coronary artery calcification and previous venous thromboembolism (File S6).

Across all three disease categories, overall marker recovery followed a consistent hierarchy across both timsTOF HT and Orbitrap Exploris 480 platforms, with Seer nanoparticle technology (Seer XT and Seer ONE) identifying the largest number of markers, followed by Top14 and PreOmics, while IGD, SP3 and IPA/TCA yielded lower coverage (Figure 3). Instrumental differences were largely quantitative, as the relative ranking of preparation methods remained conserved. A core subset of markers was consistently shared across all preparation strategies per instrument (Supporting Figure S6).

**Figure 3:**
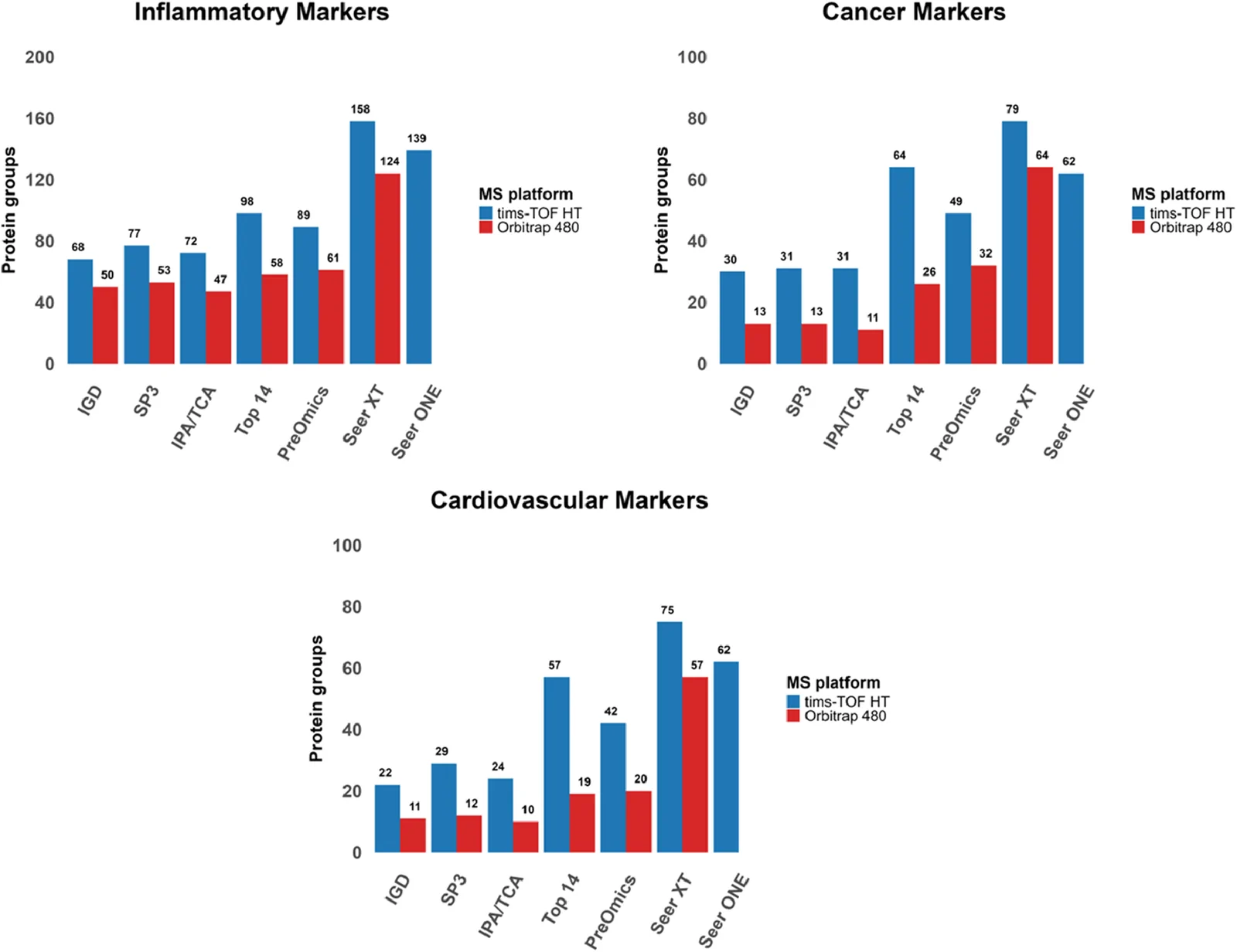
Number of identified proteins corresponding to curated inflammatory (*n* = 557), cancer (*n* = 213) and cardiovascular (*n* = 166) marker panels across all serum preparation workflows on the Orbitrap Exploris 480 and timsTOF HT platforms.

Characterization of disease-relevant protein coverage independent of individual marker panels was obtained by combining inflammatory, cancer and cardiovascular marker lists and the total number of unique markers identified by each method was calculated separately per instrument (Figure 4A). Proteins belonging to multiple categories were annotated accordingly, allowing assessment of unique marker classification and overlap between inflammatory, cancer and cardiovascular classifications. Low-depth workflows (IGD, SP3 and IPA/TCA) did not contribute to the identification of unique disease-specific marker proteins (only one unique marker detected by IPA/TCA on timsTOF HT), indicating that their detectable markers represented subsets of proteins also captured by deeper workflows, while PreOmics contributed only a limited number of unique identifications, detecting in total 4 and 1 markers in timsTOF HT and Orbitrap Exploris 480, respectively. In contrast, Top 14 yielded increased identifications, contributing 32 (timsTOF HT) and 12 (Orbitrap Exploris 480) unique markers.

**Figure 4:**
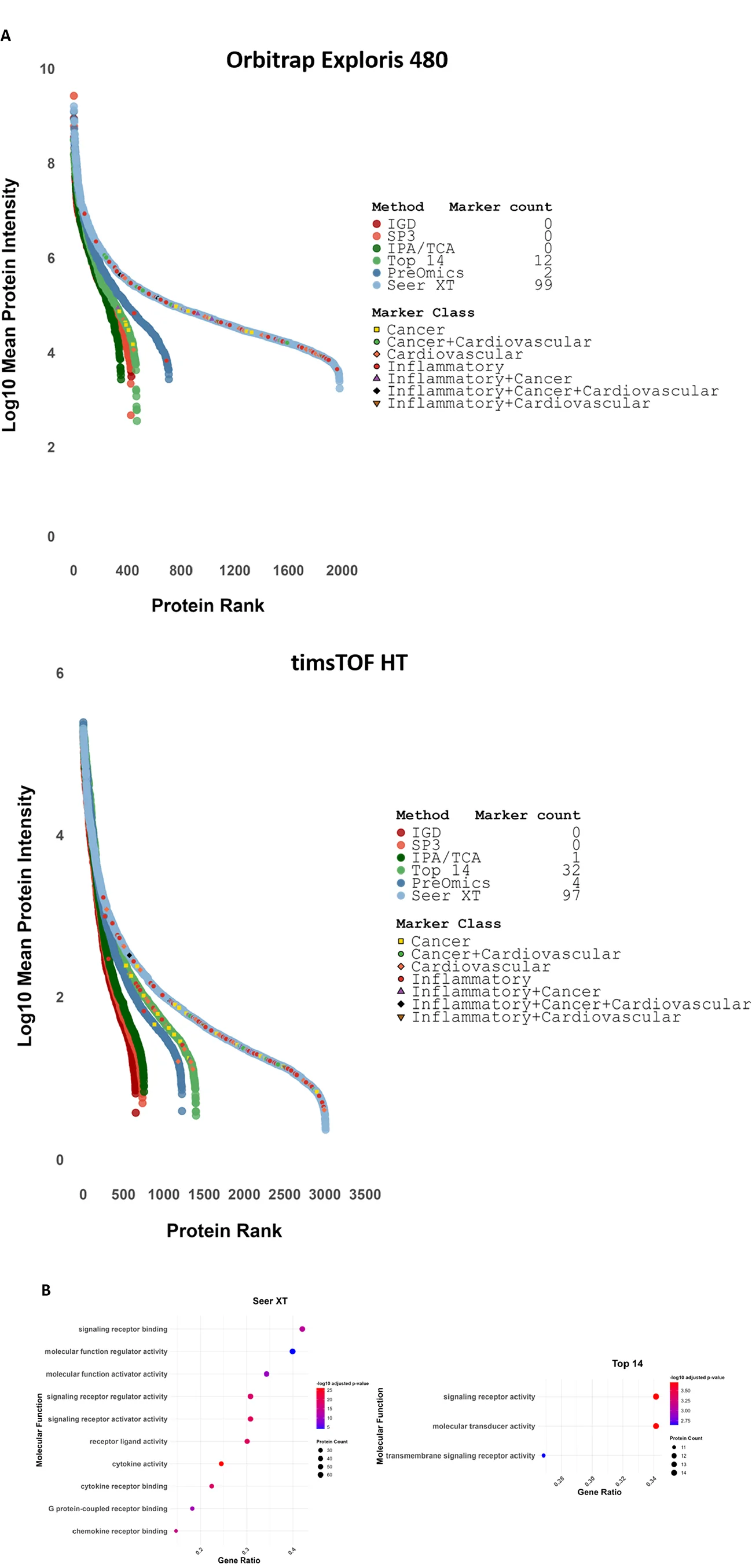
(A) Number and distribution of unique inflammatory, cancer and cardiovascular markers across all serum sample preparation methods on the Orbitrap Exploris 480 and timsTOF HT platforms. (B) Top enriched GO Molecular Function terms derived from the combined set of inflammatory, cancer and cardiovascular marker proteins identified by the Seer XT and Top 14 workflows. For each workflow, protein groups detected across both MS platforms were merged and duplicates removed prior to enrichment analysis.

Seer XT accounted for the vast majority of sample preparation method-specific proteins across all disease categories on both instruments, identifying 97 and 99 unique markers on timsTOF HT and Orbitrap Exploris 480, respectively. This substantial increase was consistent across instruments and exceeded all other preparation methods by a wide margin. As the Seer ONE workflow was analyzed exclusively on the timsTOF HT system, it was not included in the marker distribution analysis performed on each instrument. However, comparative analysis between the combined marker panels detected by Seer XT and Seer ONE showed once again a significant overlap (207 markers), with Seer XT detecting 63 and Seer ONE 23 unique markers, respectively (File S7).

Molecular function enrichment analysis of Top 14 unique markers (combined across instruments; *n* = 42) and Seer XT unique markers (combined across instruments; *n* = 143) was performed to investigate whether sample preparation strategies influence access to clinically relevant protein classes across the marker panels (Figure 4B). Analysis of Seer XT-specific marker proteins revealed a strong enrichment for signaling-associated functions. The most significantly enriched molecular functions included signaling receptor binding, receptor ligand activity, cytokine activity, cytokine receptor binding and chemokine-related activities. This suggests that the increased analytical depth provided by nanoparticle-based enrichment facilitates access to low-abundance signaling proteins that are often challenging to detect in complex serum samples. In contrast, Top14-specific marker proteins, consistent with the global enrichment analysis, were enriched primarily for receptor activity and molecular transducer functions, suggesting preferential accessibility of receptor-associated proteins.

## Conclusions

Across all evaluated workflows, timsTOF HT MS platform substantially improved analytical performance, increasing proteome depth, reducing missing values and enhancing quantitative performance. However, these gains did not redefine the relative performance of sample preparation strategies. Instead, the data demonstrate that while continued advances in MS technology are expected to further improve analytical sensitivity and coverage, instrument performance alone remains constrained by the fundamental complexity and dynamic range of the serum proteome.

Critically, this study demonstrates that sample preparation is not merely a technical pre-analytical step but a major determinant of the accessible serum proteome by shaping the composition of proteins measured by MS. Distinct preparation strategies introduced reproducible and platform-independent functional biases. Nanoparticle-based enrichment with Seer XT expanded access to a broader range of serum proteins and preferentially improved detection of clinically relevant signaling-associated proteins, including cytokine- and chemokine-related marker classes. In contrast, depletion-based preparation accessed a different subset of proteins enriched for receptor- related functions. These differences were consistently observed across MS platforms, demonstrating that they reflect intrinsic properties of the sample preparation strategies rather than differences in instrument performance.

Taken together, these findings highlight that improvements in MS instrumentation and advances in sample preparation should be considered complementary rather than interchangeable strategies for expanding biological insight from serum proteomics. Although emerging MS technologies may continue to improve analytical metrics, the ability to access specific regions of the serum proteome remains strongly influenced by sample preparation choices, particularly for low-abundance proteins affected by the extreme dynamic range of serum. Therefore, integrating appropriate sample preparation strategies with continued instrument development will be essential for maximizing the detection and quantification of biomarker-relevant proteins and for guiding workflow selection according to specific biological questions, such as the investigation of disease-associated proteins and molecular pathways.

## Supplementary Material

















## Data Availability

The mass spectrometry proteomics raw data generated by the timsTOF HT (.d files) as well as the files with the annotated spectra (.sne files) from both MS platforms have been deposited to the ProteomeXchange Consortium via the PRIDE partner repository with the data set identifier PXD078563. The mass spectrometry proteomics raw data generated by the Orbitrap Exploris 480 (.raw files) have been previously stored with the data set identifier PXD062785.

## Notes

Reagents and Instrumentation, missing values (Figure S1), protein group overlap (Figure S2), protein group variation (Figure S3), linearity of spiked-in proteins (Figure S4), GO analysis per MS platform (Figure S5) and overlaps of disease markers across all workflows and MS platforms (Figure S6); concentrations of spiked-in proteins (Table S1), average number of peptides per protein group (Table S2), median peptide yield (Table S3), peak capacities (Table S4), variance of spiked-in proteins-HT (Table S5), variance of spiked-in proteins-480 (Table S6), endogenous concentrations of spiked-in proteins-HT (Table S7), endogenous concentrations of spiked-in proteins-480 (Table S8), peptide coverage of spiked-in proteins-HT (Table S9), peptide coverage of spiked-in proteins-480 (Table S10), and precursor-level median CV% (Table S11) (PDF)
Protein groups per method for both MS platforms (File S1) (XLSX)
Precursors per method for both MS platforms (File S2) (XLSX)
Unique precursor features on each MS platform (File S3) (XLSX)
Intensity ratios of spiked-in proteins for Seer XT (File S4) (XLSX)
GO analysis on timsTOF HT unique protein groups (File S5) (XLSX)
Lists of marker panels (File S6) (XLSX)
Shared and unique markers between Seer XT and Seer ONE (File S7) (XLSX)
