# Supplementary material for: Cross-Platform Evaluation Reveals Sample Preparation-Driven Biological Biases despite Advances in Mass Spectrometry Performance in Serum Proteomics
Source: J Proteome Res. 2026 Sep 10;25(10):5480–92. doi: 10.1021/acs.jproteome.6c00493 (PMC13640508; doi:10.1021/acs.jproteome.6c00493)
Supplement: Supplementary file 1 [file pr6c00493_si_001.pdf]

Supporting Information

Cross-Platform Evaluation Reveals Sample Preparation-Driven Biological Biases Despite Advances in Mass Spectrometry Performance in Serum Proteomics

Drosos Katsavelis*[1,2], Marieke G.C. van der Hart[1,2], Justina C. Wolters[3], Hjalmar P. Permentier[2], Peter L. Horvatovich[2], Thomas I.F.H. Cremers[1,2]

[1]Quantall B.V., L.J. Zielstraweg 1, 9713 GX Groningen, The Netherlands
[2]Department of Analytical Biochemistry, University of Groningen, Antonius Deusinglaan 1, 9713 AV Groningen, The Netherlands
[3]Department of Pediatrics, University of Groningen, University Medical Center Groningen, Antonius Deusinglaan 1, 9713 AV Groningen, The Netherlands
*Author correspondence to drosos.katsavelis@quantall.com

Table of Contents

List of Reagents and instrumentation S2
Supporting Figures S3
Supporting Figure S1: Missing values S3
Supporting Figure S2: Protein group overlap S3
Supporting Figure S3: Protein group variation S4
Supporting Figure S4: Linearity of spiked-in proteins S5
Supporting Figure S5: GO analysis per MS platform S12
Supporting Figure S6: Overlaps of disease markers across all workflows and MS platforms S13
Supporting Tables S16
Supporting Table S1: Concentrations of spiked-in proteins S16
Supporting Table S2: Average number of peptides per protein group S16
Supporting Table S3: Median peptide yield S17
Supporting Table S4: Peak capacities S17
Supporting Table S5: Variance of spiked-in proteins-HT S17
Supporting Table S6: Variance of spiked-in proteins-480 .... S18
Supporting Table S7: Endogenous concentrations of spiked-in proteins-HT S18
Supporting Table S8: Endogenous concentrations of spiked-in proteins-480 .... S18
Supporting Table S9: Peptide coverage of spiked-in proteins-HT S19
Supporting Table S10: Peptide coverage of spiked-in proteins-480 .... S19
Supporting Table S11: Precursor-level median CV% S19
Supporting Files
File S1: Protein groups per method for both MS platforms (XLSX)
File S2: Precursors per method for both MS platforms (XLSX)
File S3: Unique precursor features on each MS platform (XLSX)
File S4: Intensity ratios of spiked-in proteins for Seer XT (XLSX)
File S5: GO analysis on timsTOF HT unique protein groups (XLSX)
File S6: Lists of marker panels (XLSX)
File S7: Shared and unique markers between Seer XT and Seer ONE (XLSX)

Reagents and instrumentation

NuPage LDS-Sample buffer (4X), gel electrophoresis (SDS-PAGE) 4–12% Bis-Tris 15-wells-gel, Pierce Quantitative Colorimetric Peptide Assay, High Select Top14 Abundant Protein Depletion Resin mini columns, and Pierce HeLa Protein Digest Standard were purchased by Thermo Fisher Scientific; ammonium bicarbonate (ABC), DL-dithiothreitol (DTT), iodoacetamide (IAA), formic acid (FA), trichloroacetic acid (TCA), and sodium deoxycholate (SDC) from Sigma-Aldrich; Rapid Protein Stain Coomassie Blue from Westburg; acetonitrile (ACN), isopropanol (IPA), absolute methanol, and absolute ethanol from Biosolve; sequencing grade-modified trypsin (V5111) from Promega; ENRICH-iST Kits 8x from PreOmics; Sera-Mag Carboxylate-Modified Magnetic Beads (44152105050250 (E3) and 24152105050250 (E7)) from Cytiva; pooled human serum from TCS Biosciences Ltd.; Sapphire low retention pipet tips from Greiner; and pipet tips for gel loading from Bio-Rad.

Bravo Automated Liquid Handling Platform from Agilent and Alpaqua Magnum FLX Enhanced Universal Magnet Plate from Alpaqua were used for the binding and washing steps of the SP3 protocol, while the 16-Tube SureBeads Magnetic Rack from Bio-Rad was used for the PreOmics protocol. The digested peptides were dried with the Concentrator plus/Vacufuge plus from Eppendorf. NanoPhotometer N120 from Implen was used to measure the protein concentration for IPA/TCA and Top 14 protocols. SP100 Automation Instrument was used by Seer to perform the Proteograph XT protocol.

Supporting Figures

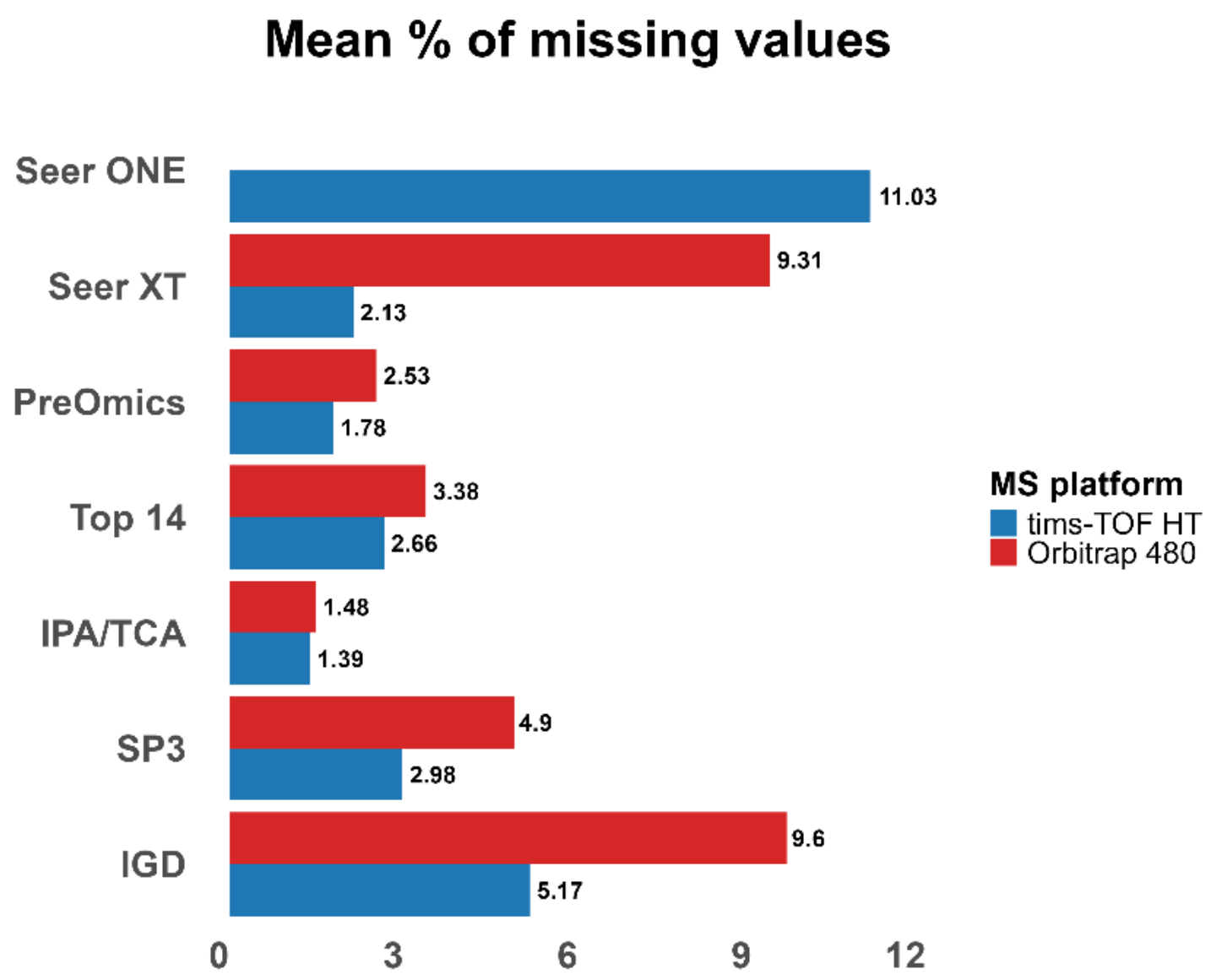

Supporting Figure S1. Percentage of average missing values per method on both MS platforms.

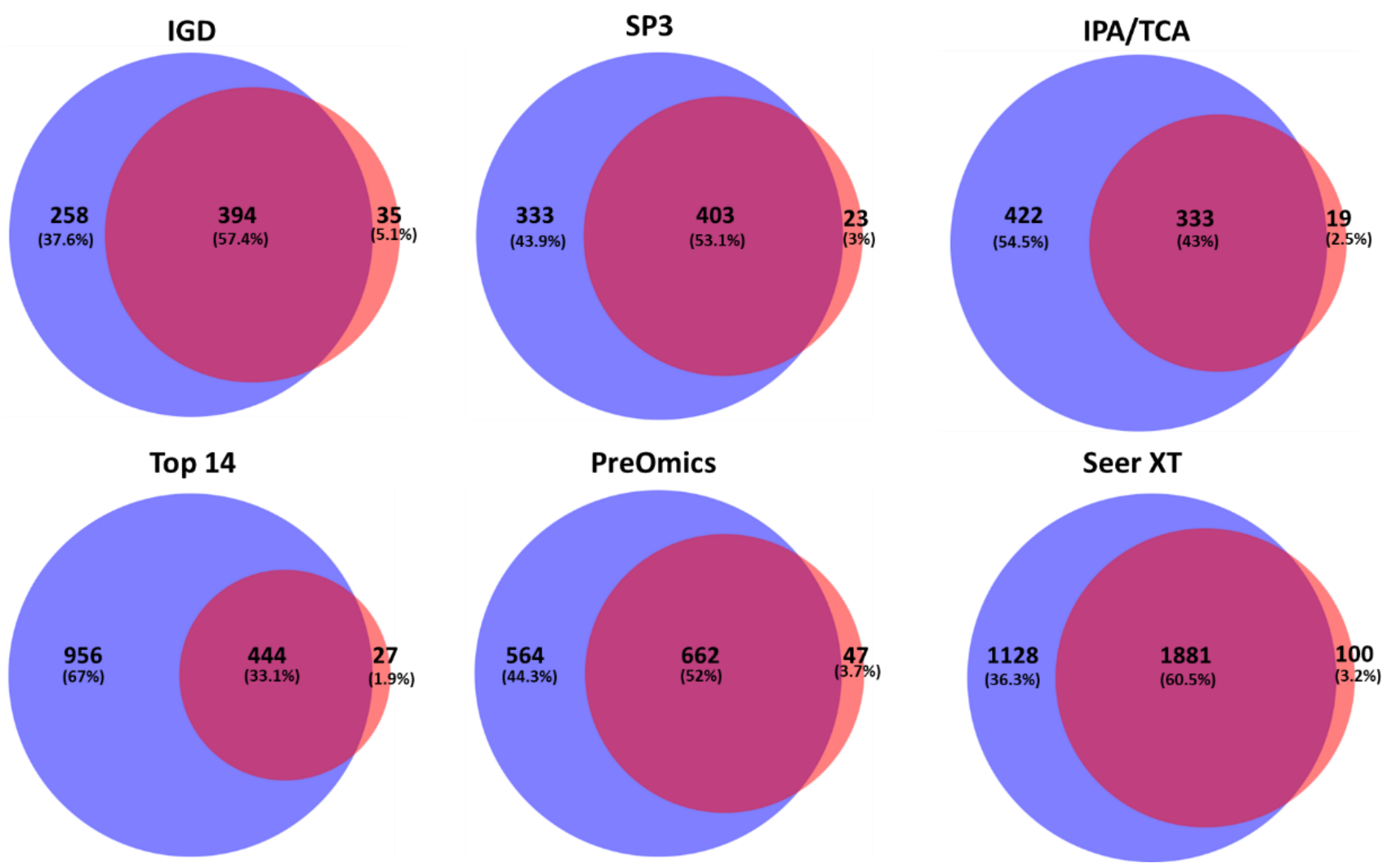

Supporting Figure S2. Protein group overlap for each method on both MS platforms.

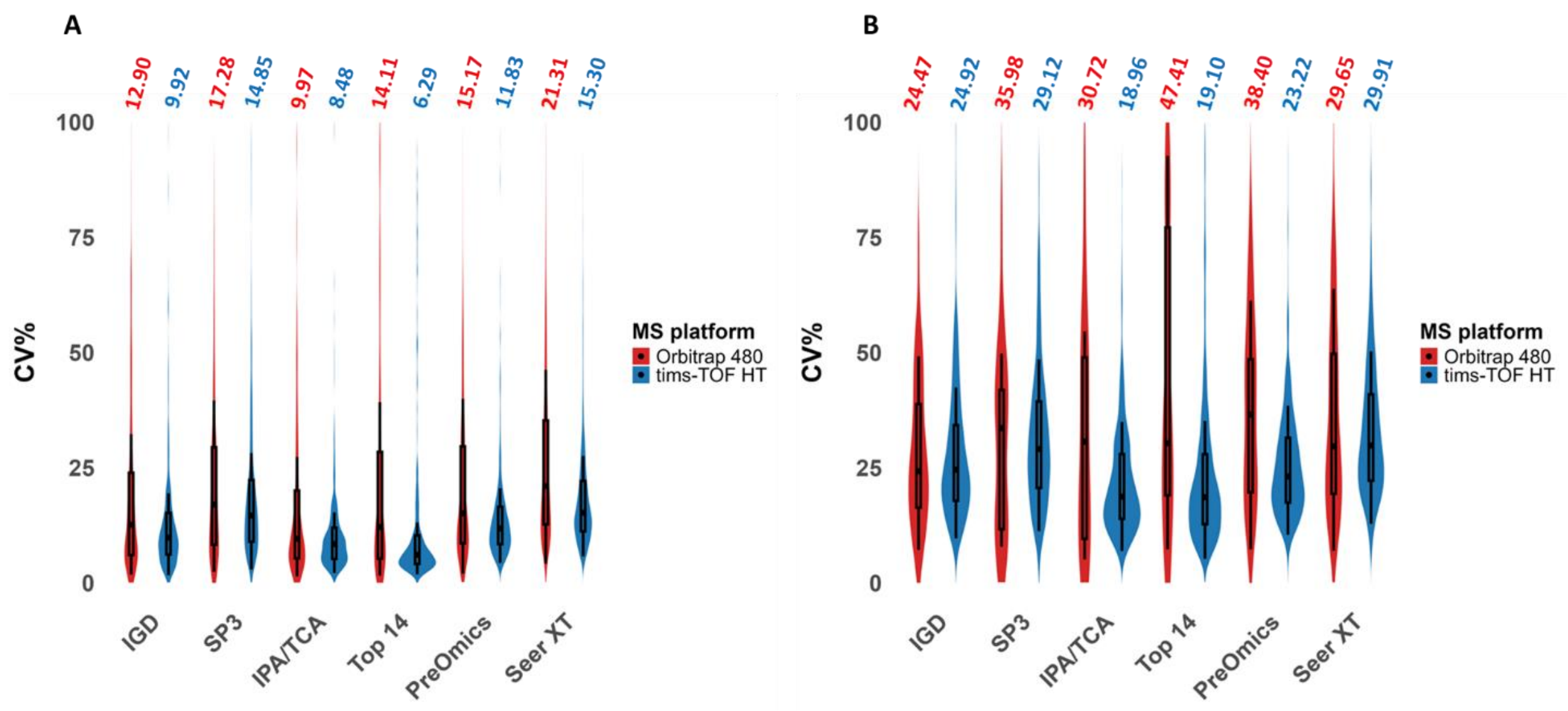

Supporting Figure S3. CV% distributions for A) shared protein groups on both MS platforms and B) unique protein groups on each MS platform.

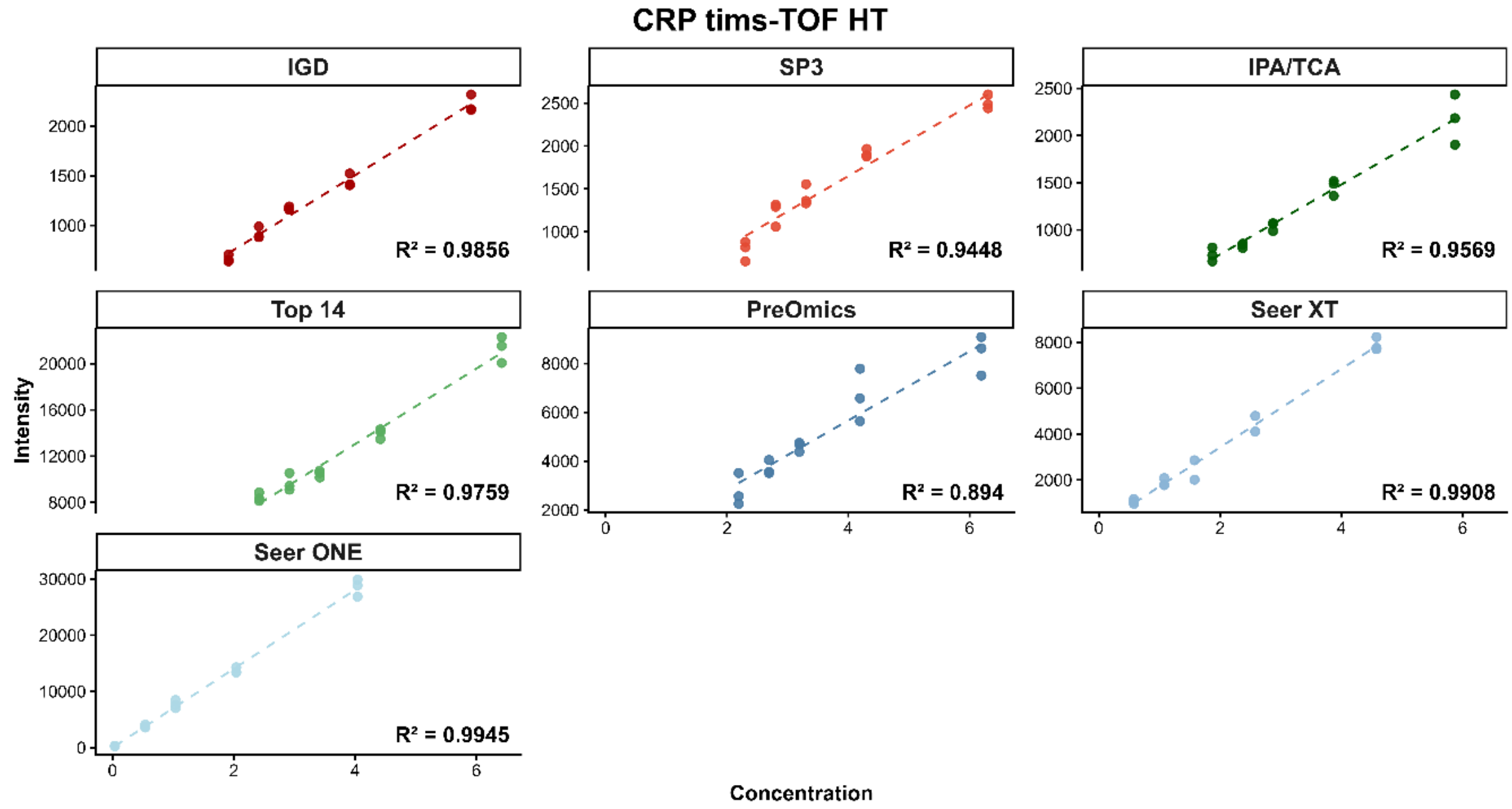
CRP tims-TOF HT
IGD
SP3
IPA/TCA
Top 14
PreOmics
Seer XT
Seer ONE
R² = 0.9856
R² = 0.9448
R² = 0.9569
R² = 0.9759
R² = 0.894
R² = 0.9908
R² = 0.9945
Intensity
Concentration

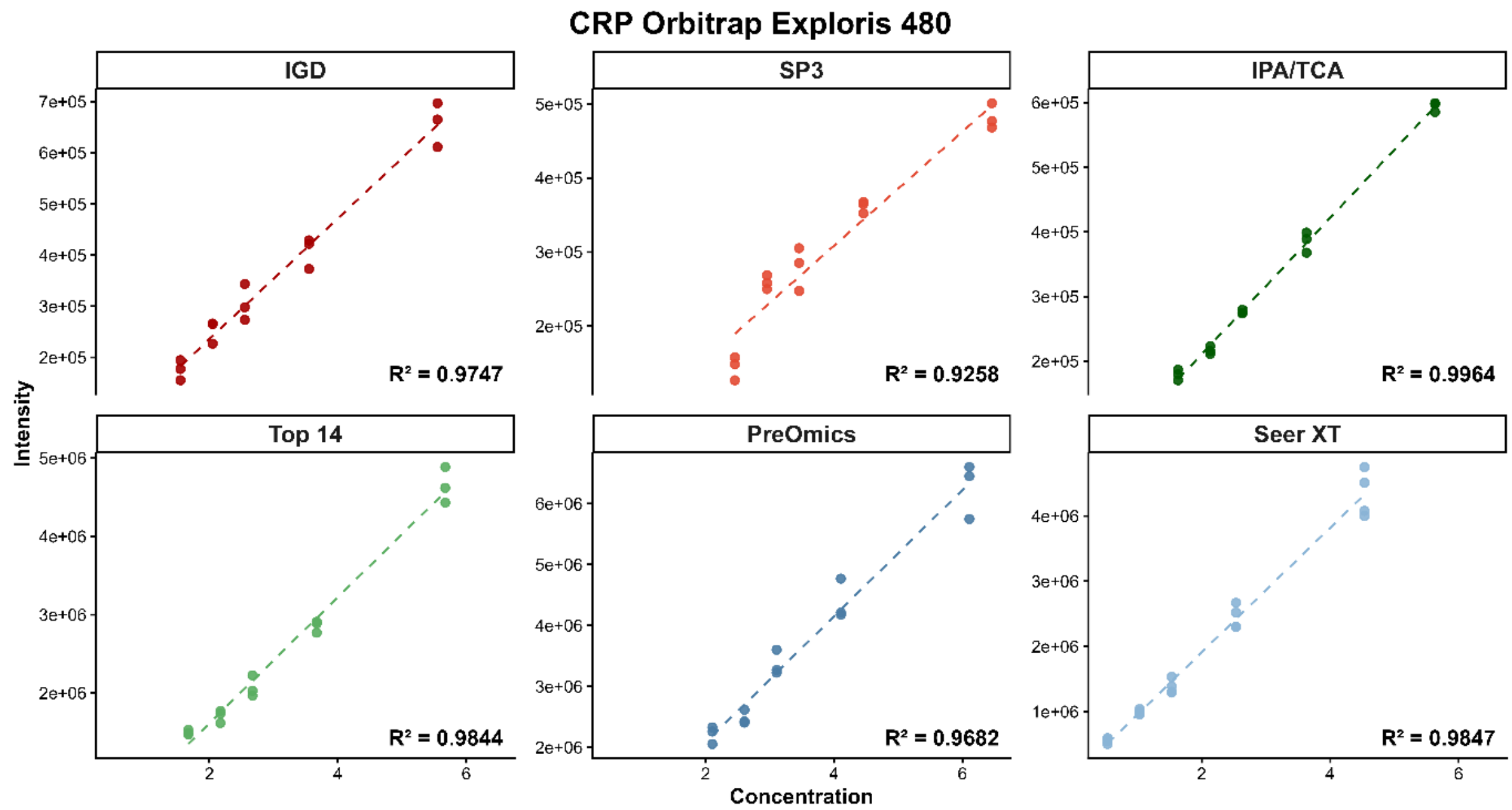
CRP Orbitrap Exploris 480
IGD
SP3
IPA/TCA
Top 14
PreOmics
Seer XT
R² = 0.9747
R² = 0.9258
R² = 0.9964
R² = 0.9844
R² = 0.9682
R² = 0.9847
Intensity
Concentration

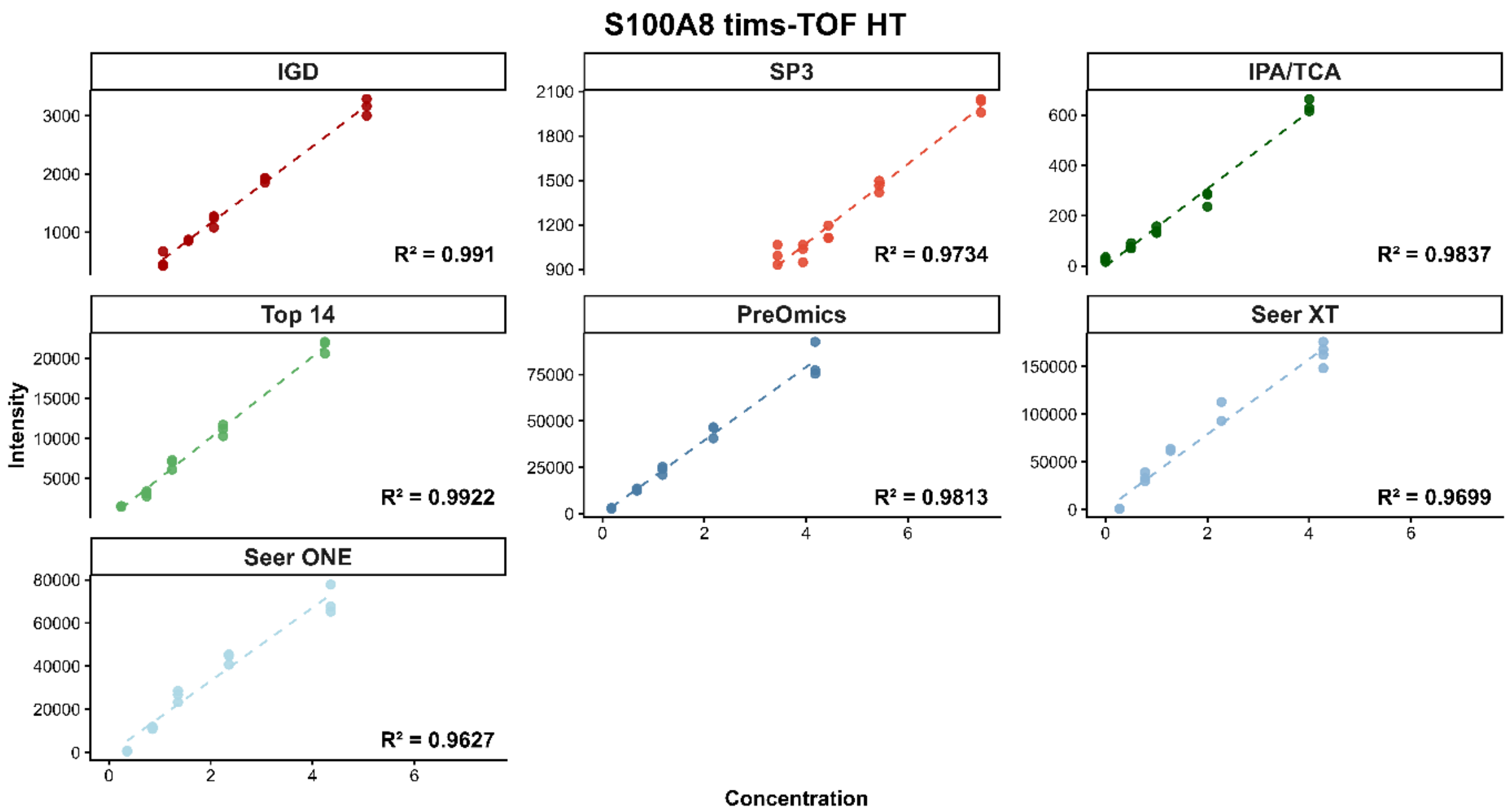
S100A8 tims-TOF HT
IGD
R² = 0.991
SP3
R² = 0.9734
IPA/TCA
R² = 0.9837
Top 14
R² = 0.9922
PreOmics
R² = 0.9813
Seer XT
R² = 0.9699
Seer ONE
R² = 0.9627
Intensity
Concentration

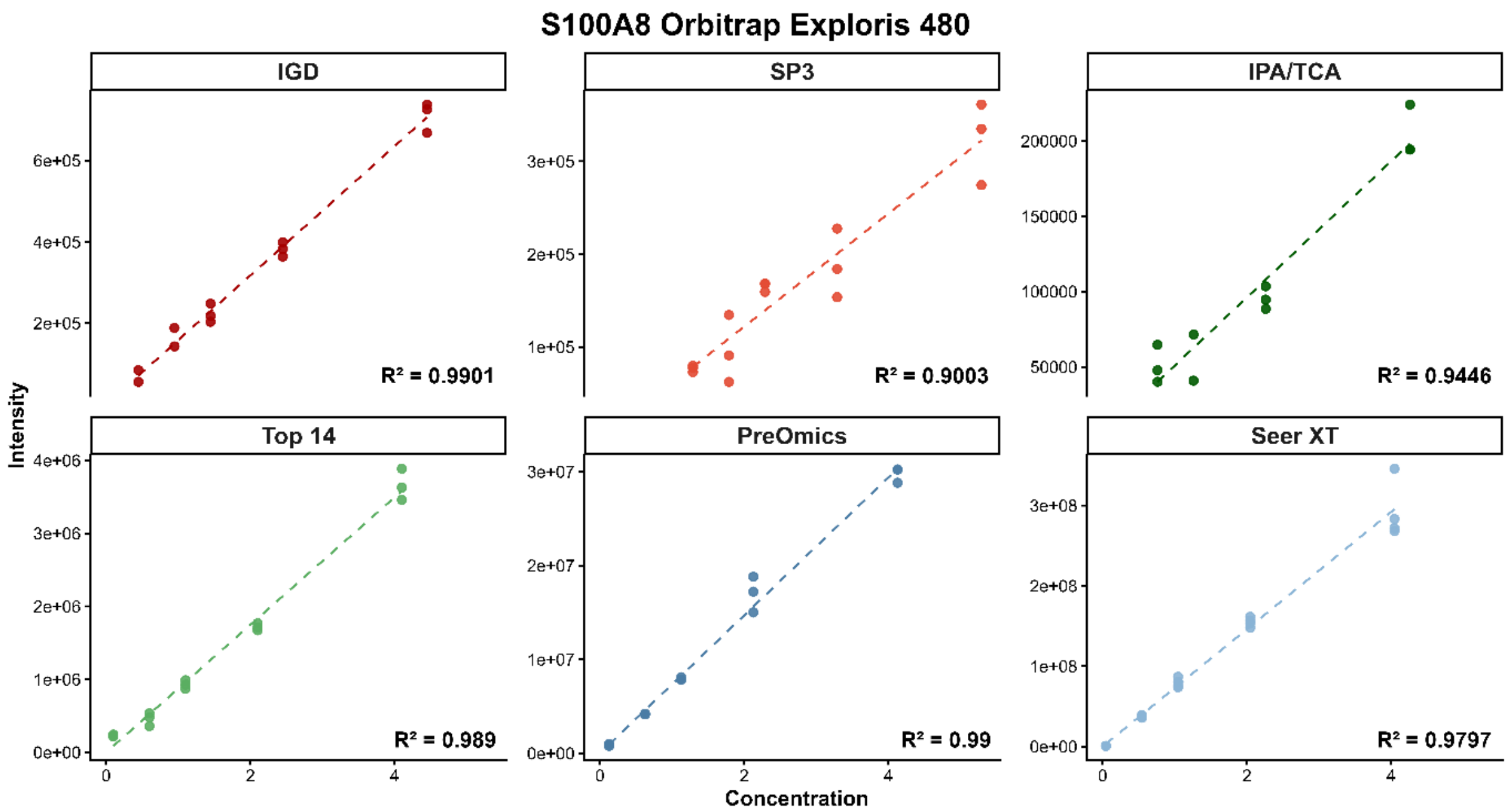
S100A8 Orbitrap Exploris 480
IGD
R² = 0.9901
SP3
R² = 0.9003
IPA/TCA
R² = 0.9446
Top 14
R² = 0.989
PreOmics
R² = 0.99
Seer XT
R² = 0.9797
Intensity
Concentration

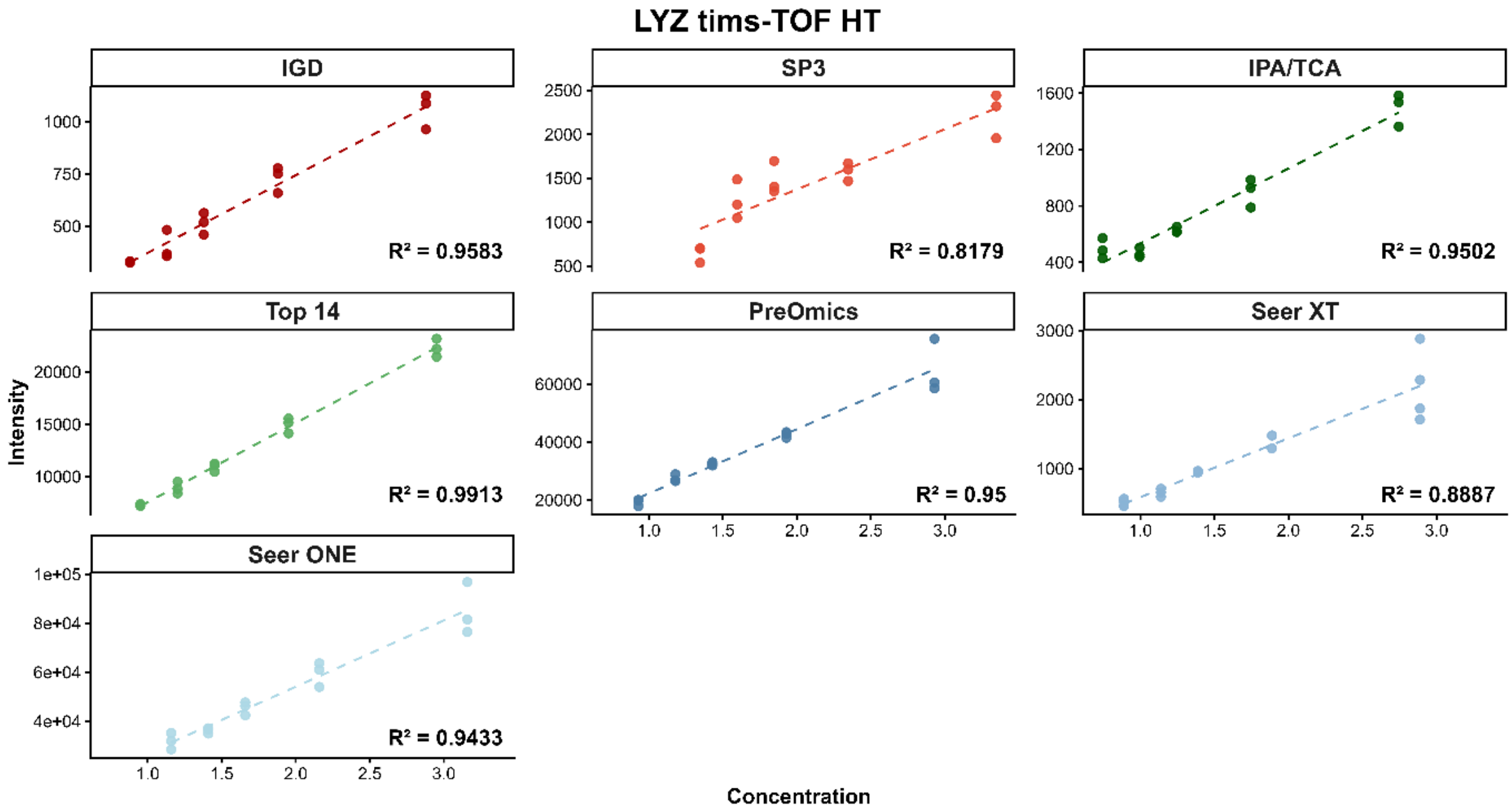
LYZ tims-TOF HT
IGD
R² = 0.9583
SP3
R² = 0.8179
IPA/TCA
R² = 0.9502
Top 14
R² = 0.9913
PreOmics
R² = 0.95
Seer XT
R² = 0.8887
Seer ONE
R² = 0.9433
Intensity
Concentration

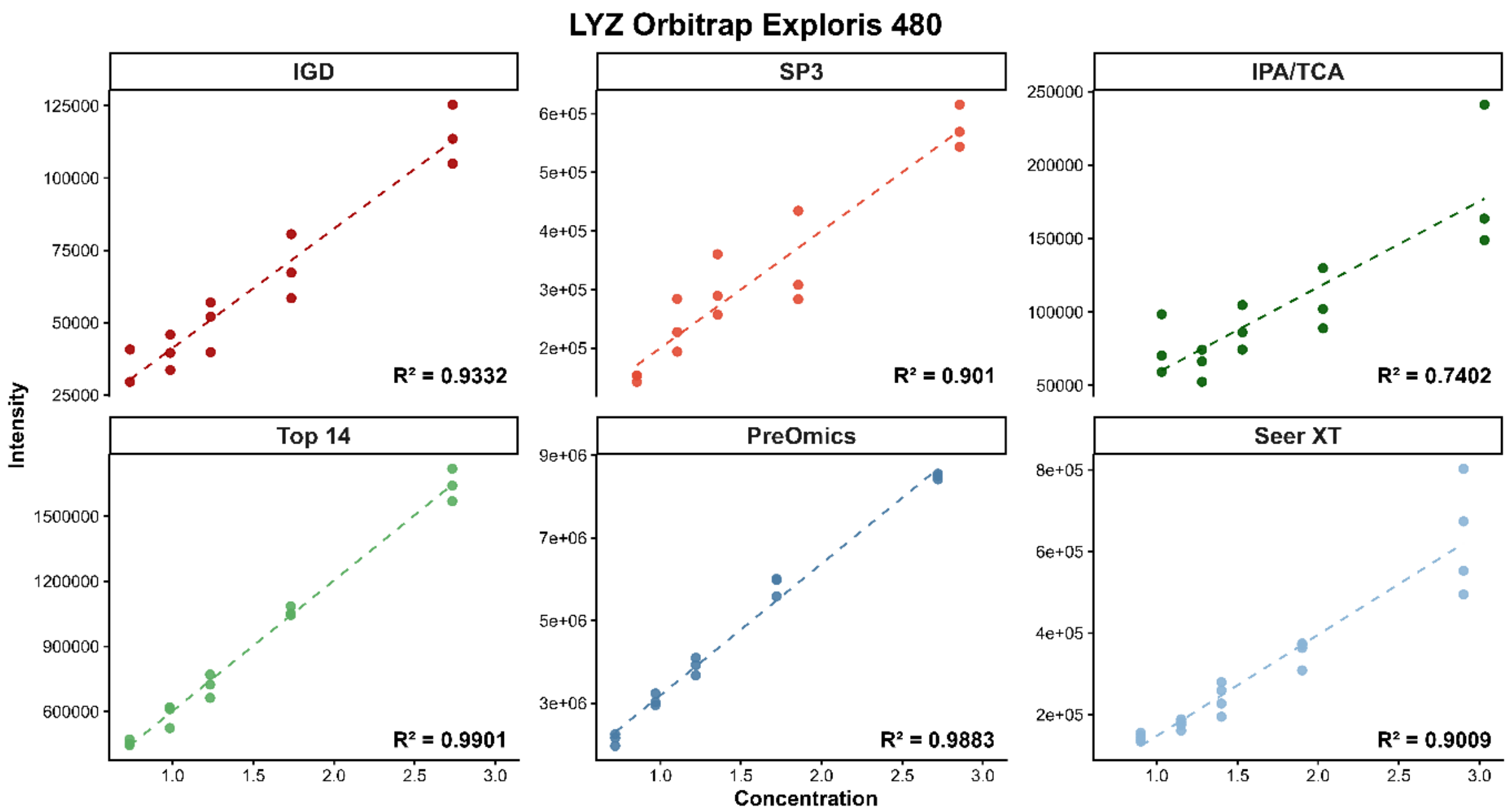
LYZ Orbitrap Exploris 480
IGD
R² = 0.9332
SP3
R² = 0.901
IPA/TCA
R² = 0.7402
Top 14
R² = 0.9901
PreOmics
R² = 0.9883
Seer XT
R² = 0.9009
Intensity
Concentration

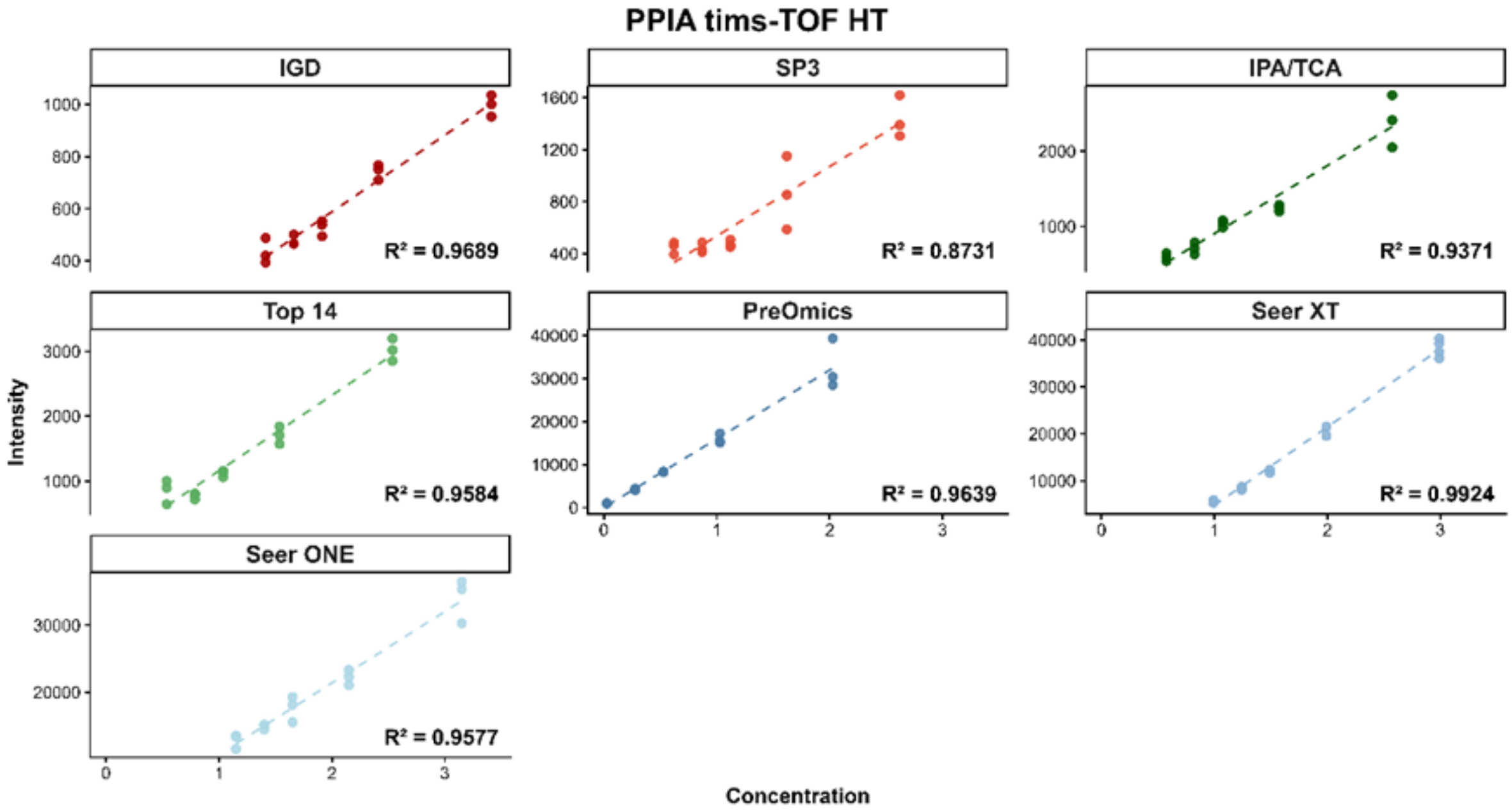
PPIA tims-TOF HT
IGD
SP3
IPA/TCA
Top 14
PreOmics
Seer XT
Seer ONE
R² = 0.9689
R² = 0.8731
R² = 0.9371
R² = 0.9584
R² = 0.9639
R² = 0.9924
R² = 0.9577
Intensity
Concentration

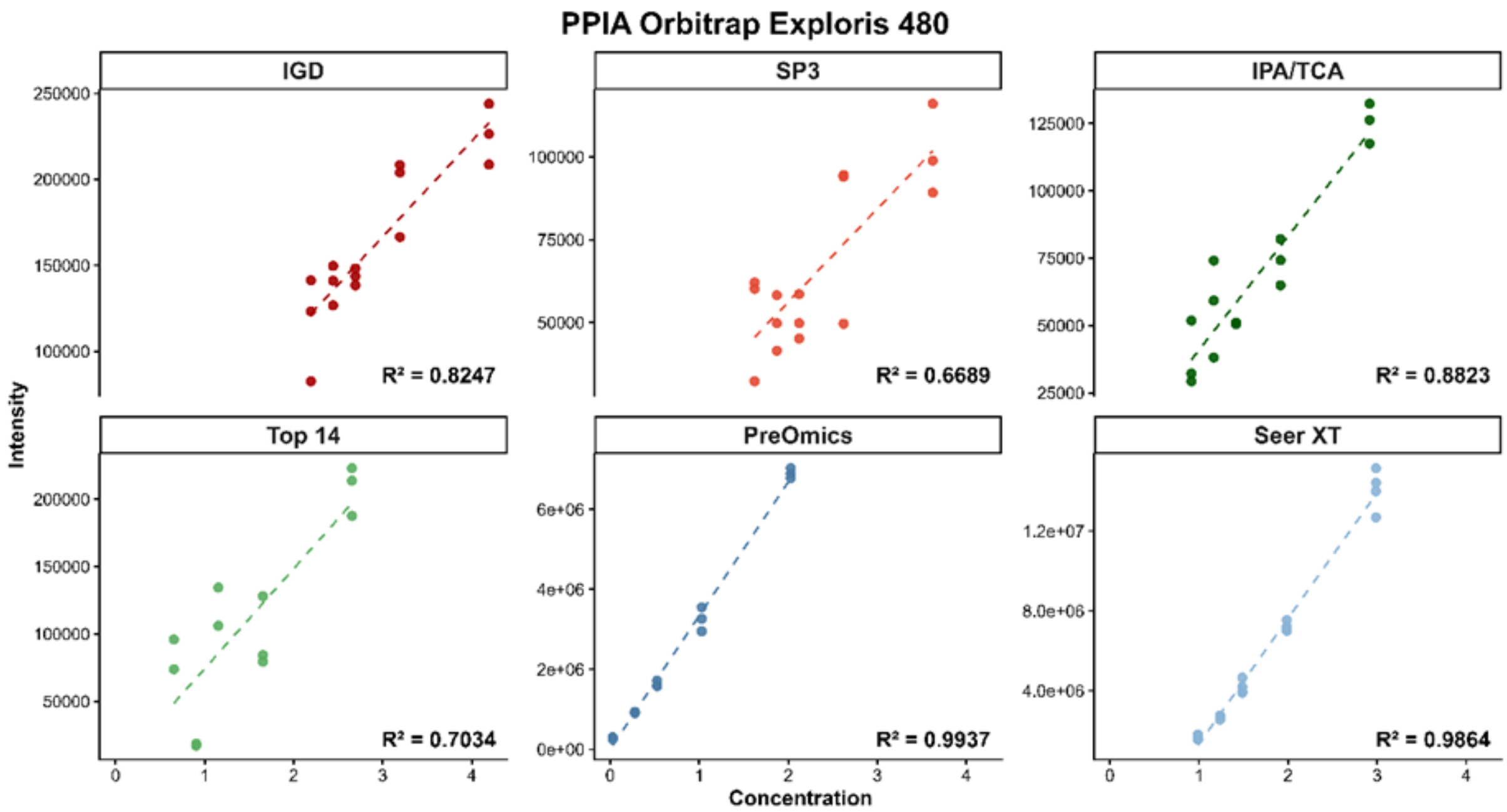
PPIA Orbitrap Exploris 480
IGD
SP3
IPA/TCA
Top 14
PreOmics
Seer XT
R² = 0.8247
R² = 0.6689
R² = 0.8823
R² = 0.7034
R² = 0.9937
R² = 0.9864
Intensity
Concentration

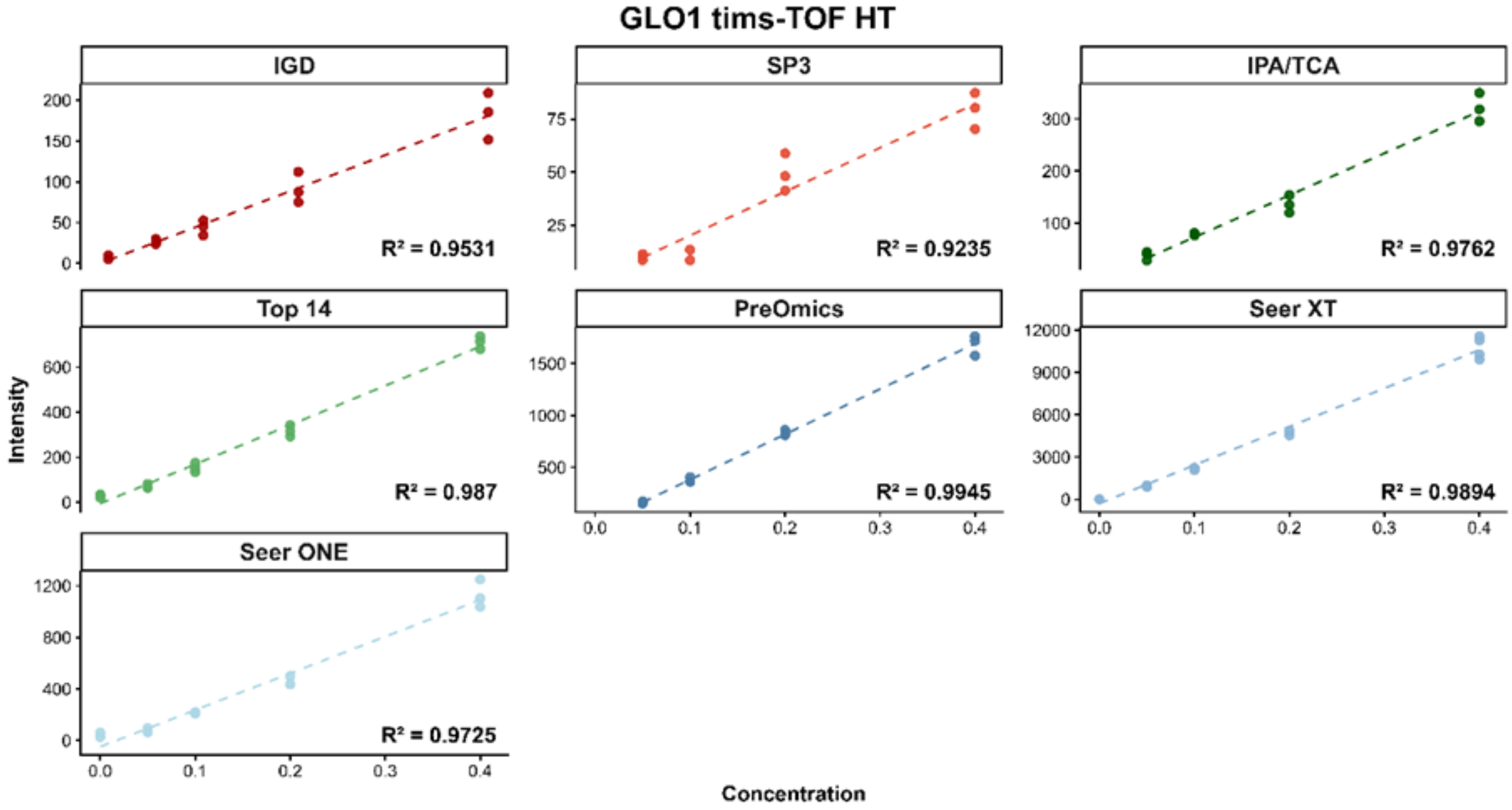
GLO1 tims-TOF HT
IGD
SP3
IPA/TCA
Top 14
PreOmics
Seer XT
Seer ONE
R² = 0.9531
R² = 0.9235
R² = 0.9762
R² = 0.987
R² = 0.9945
R² = 0.9894
R² = 0.9725
Intensity
Concentration

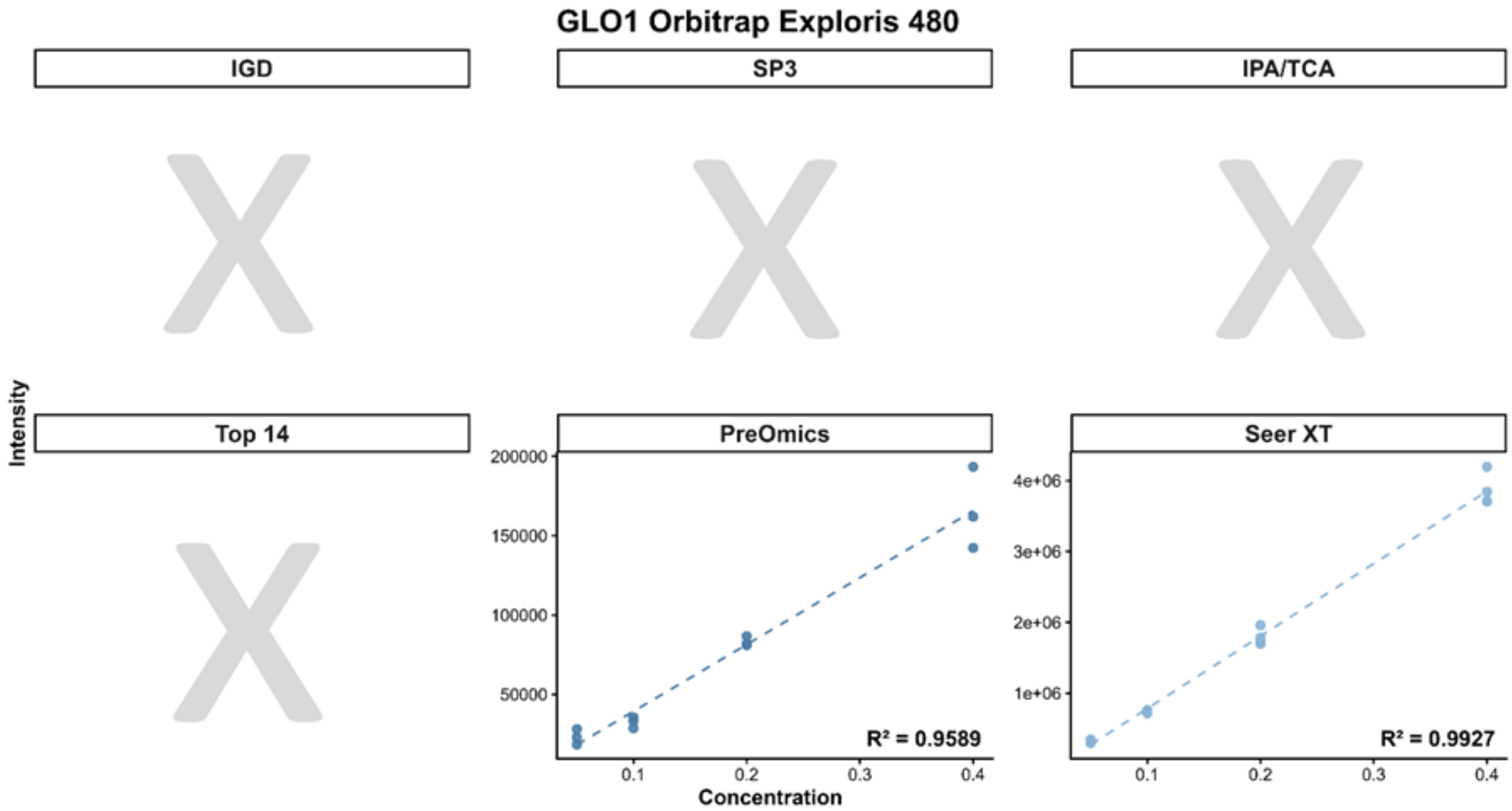
GLO1 Orbitrap Exploris 480
IGD
SP3
IPA/TCA
Top 14
PreOmics
Seer XT
R² = 0.9589
R² = 0.9927
Intensity
Concentration

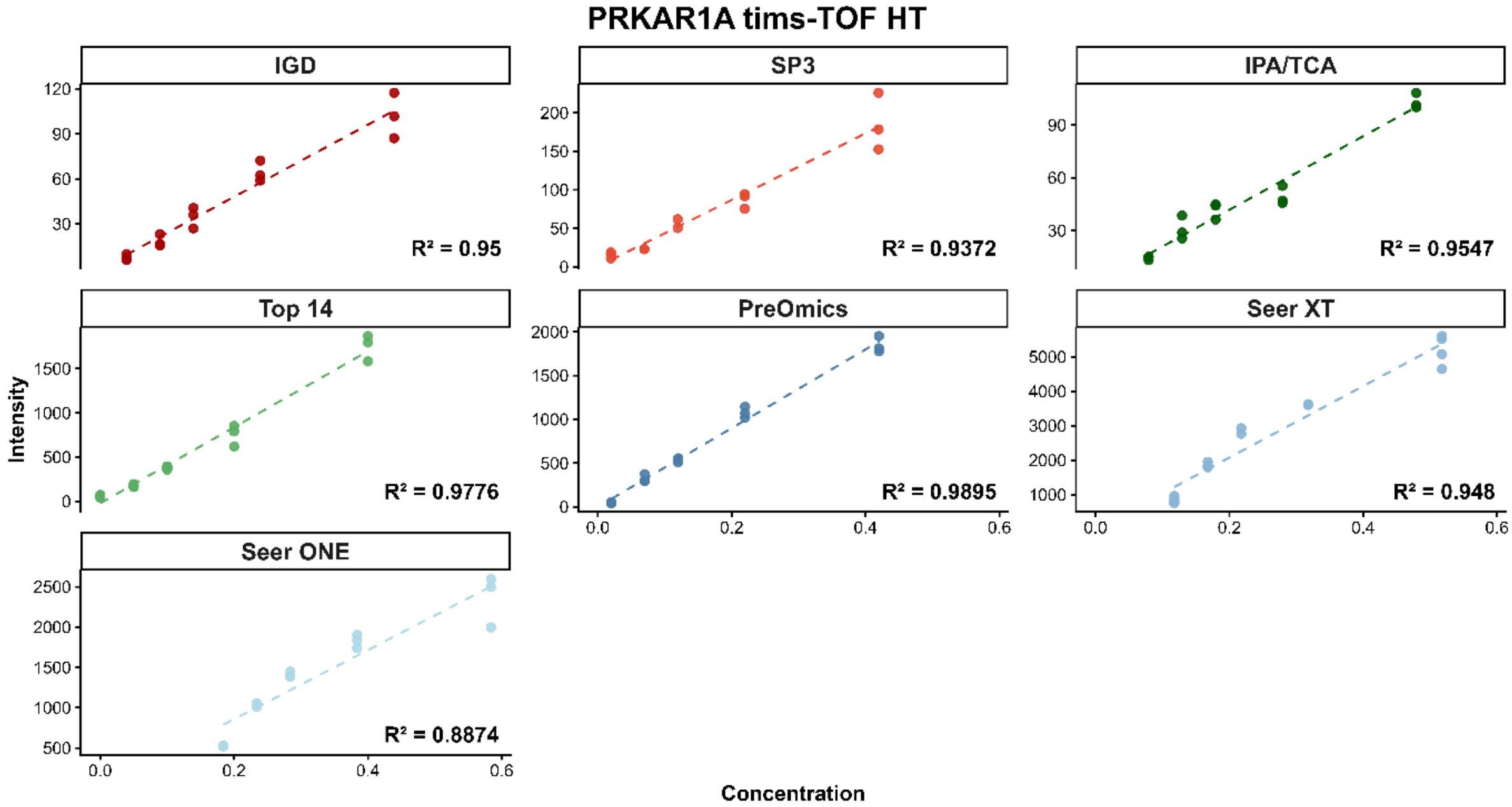
PRKAR1A tims-TOF HT
IGD
R² = 0.95
SP3
R² = 0.9372
IPA/TCA
R² = 0.9547
Top 14
R² = 0.9776
PreOmics
R² = 0.9895
Seer XT
R² = 0.948
Seer ONE
R² = 0.8874
Intensity
Concentration

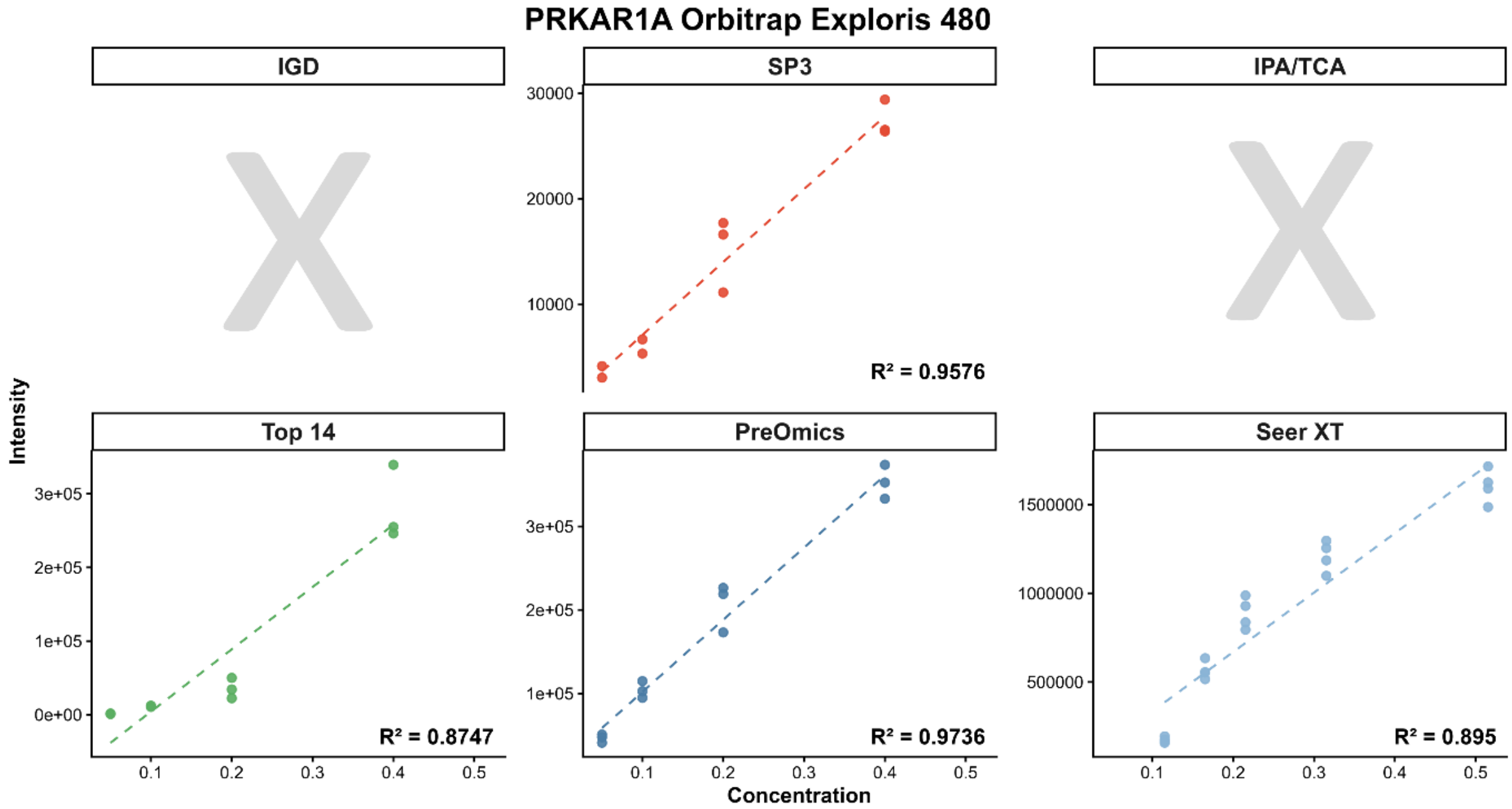
PRKAR1A Orbitrap Exploris 480
IGD
SP3
R² = 0.9576
IPA/TCA
Top 14
R² = 0.8747
PreOmics
R² = 0.9736
Seer XT
R² = 0.895
Intensity
Concentration

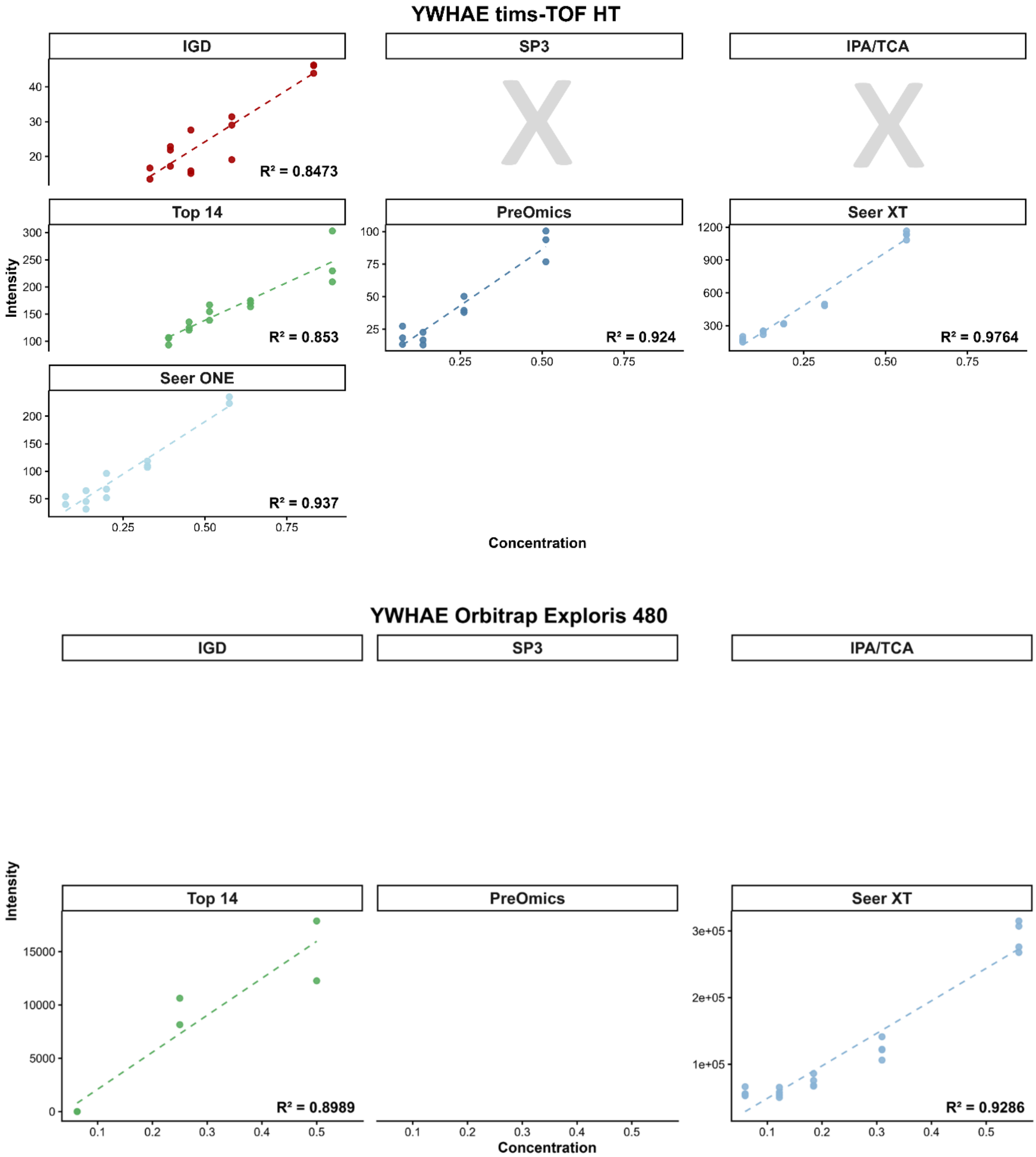

Supporting Figure S4. Linearity and variance explained by the linear model of spiked-in proteins across all sample preparation methods by plotting the protein intensities of all replicates versus the spiked concentrations (ng/µL) of CRP, S100A8, LYZ, PPIA, GLO1, PRKAR1A and YWHAE for both MS platforms. Whole proteome analysis methods are highlighted with red color, depletion methods with green color and enrichment methods with blue color.

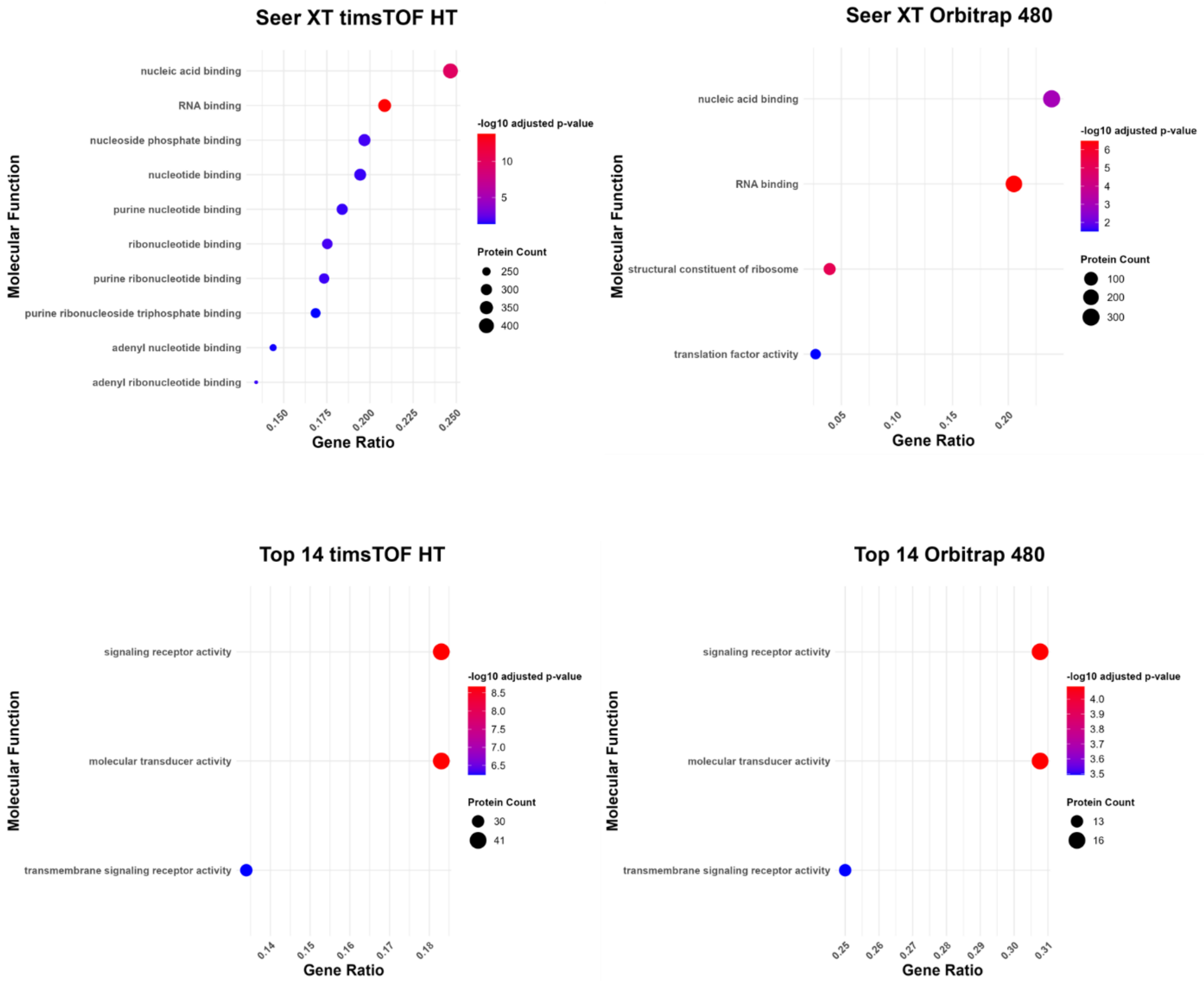

Supporting Fisure S5. Top enriched Gene Ontology (GO) Molecular Function terms derived from unique protein groups identified by the Seer XT and Top 14 workflows for timsTOF HT and Orbitrap Exploris 480.

Inflammatory markers-Orbitrap Exploris 480

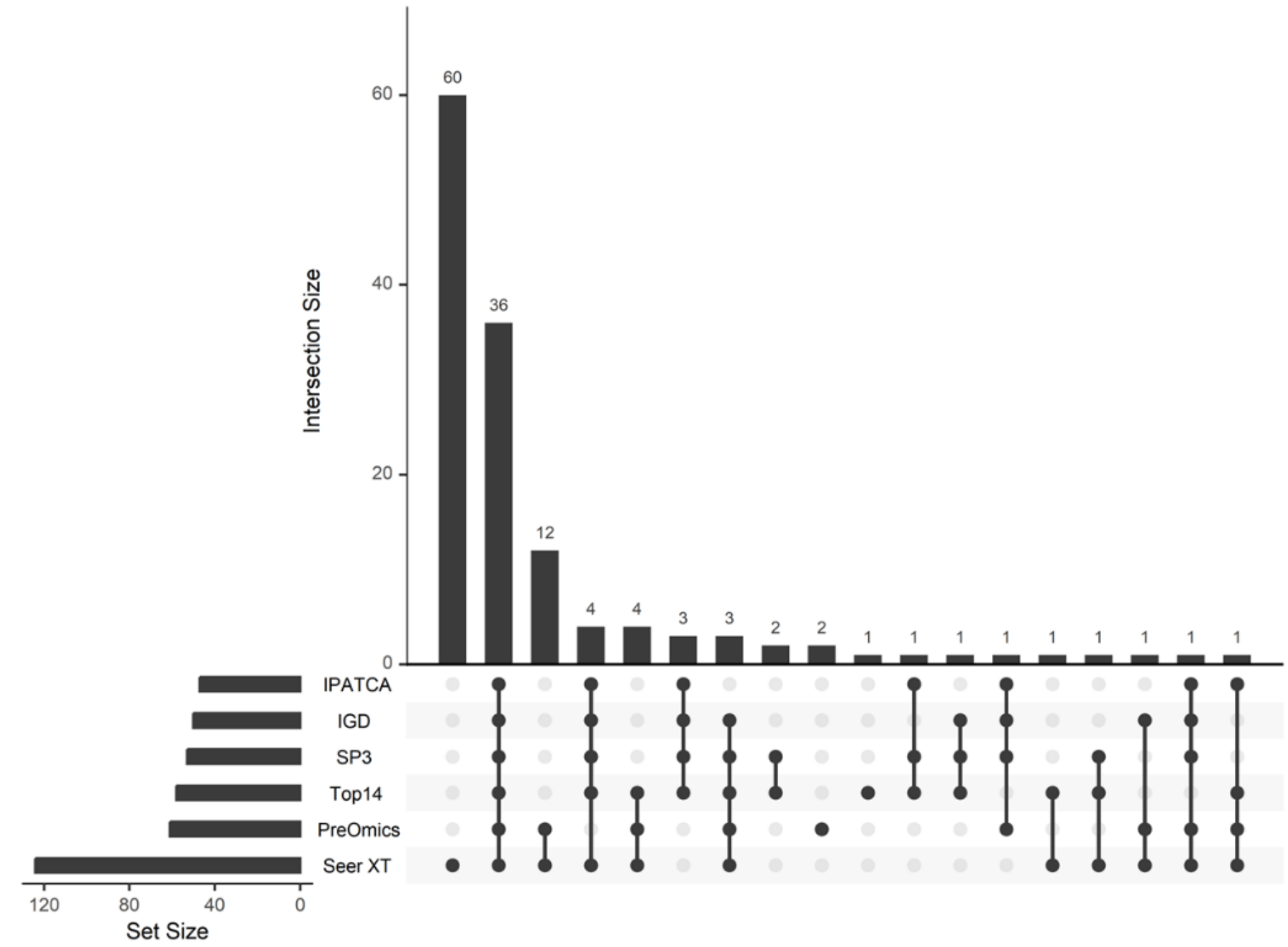

Inflammatory markers-timsTOF HT

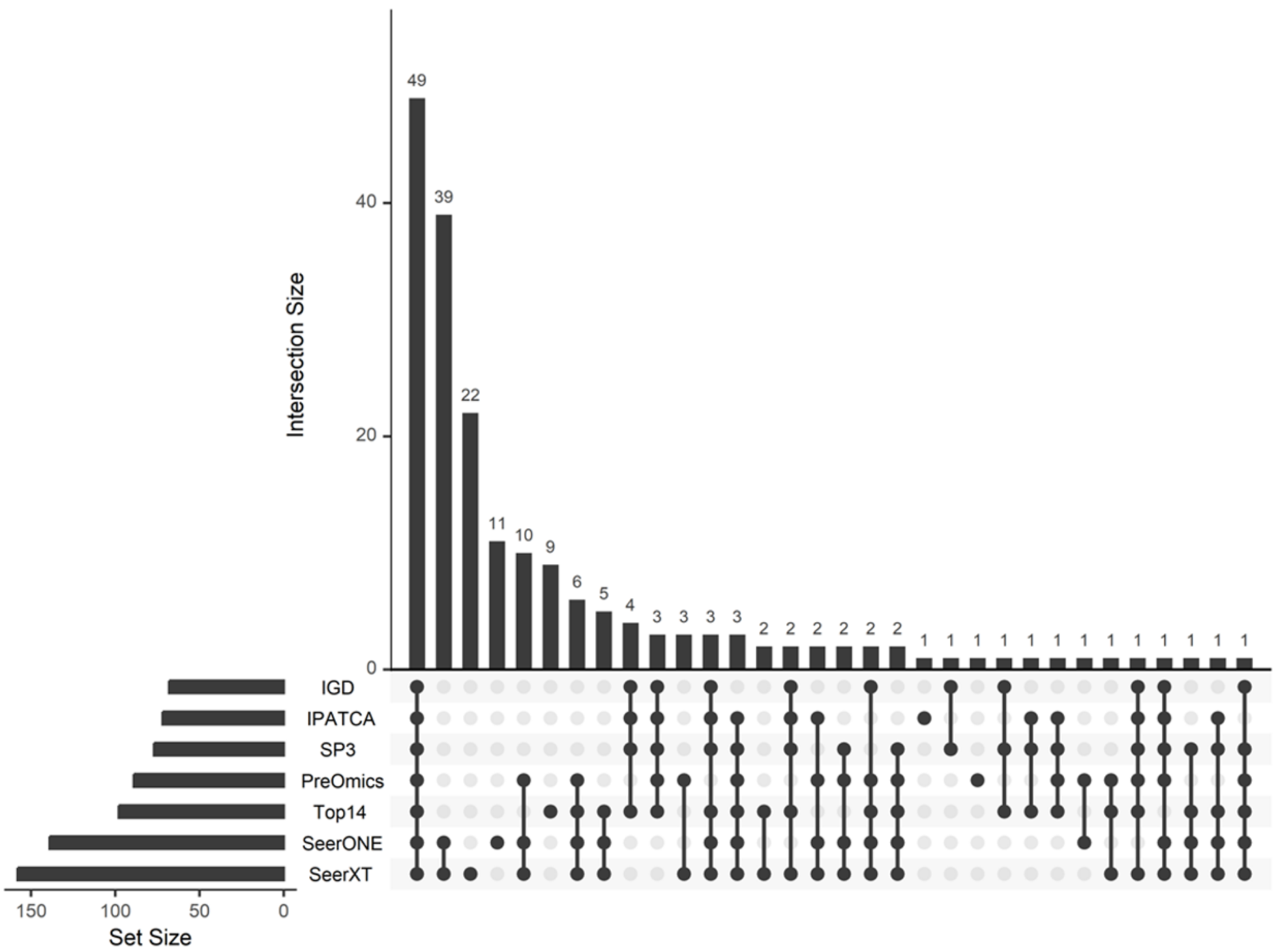

Cancer markers-Orbitrap Exploris 480

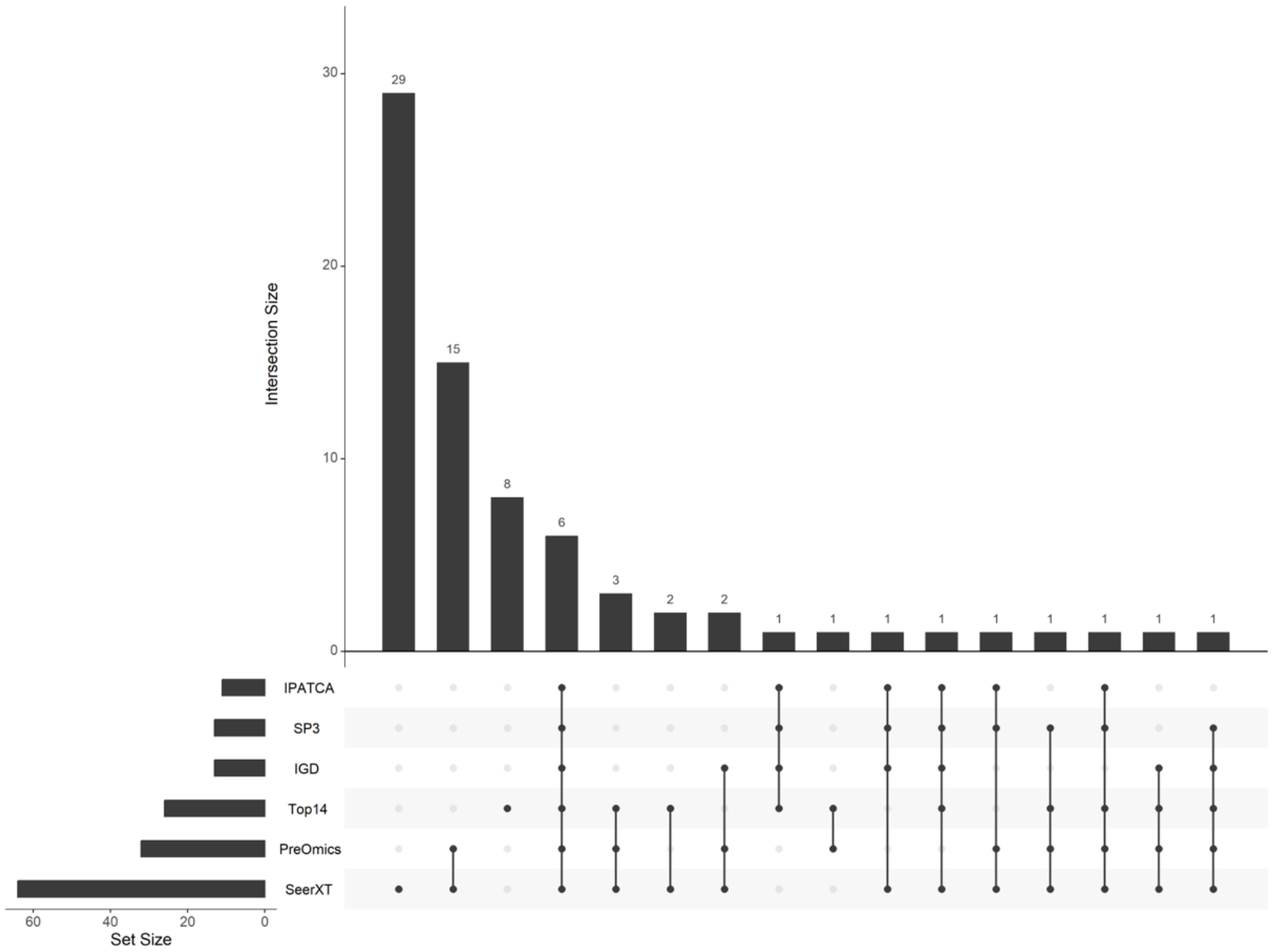

Cancer markers-timsTOF HT

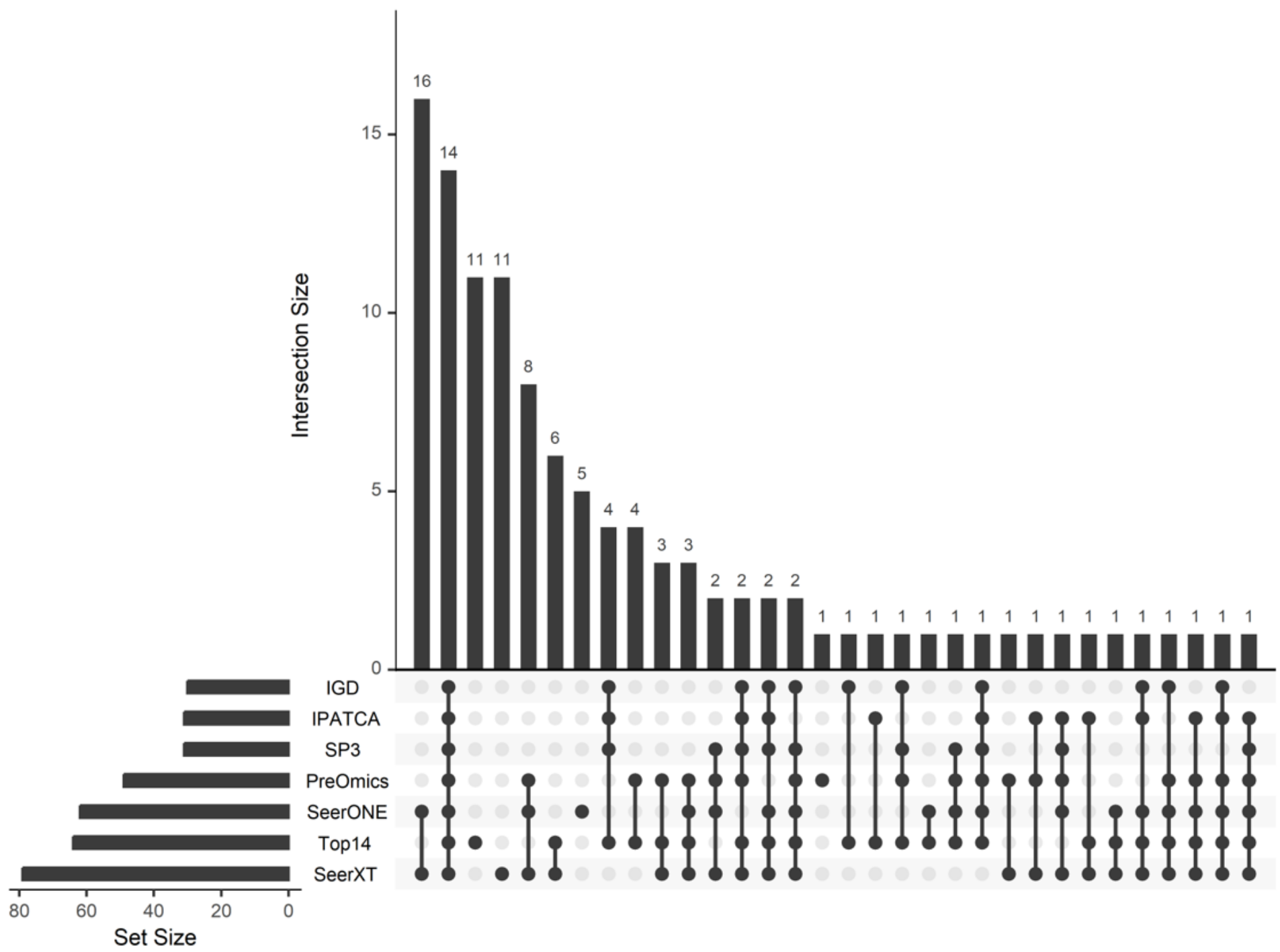

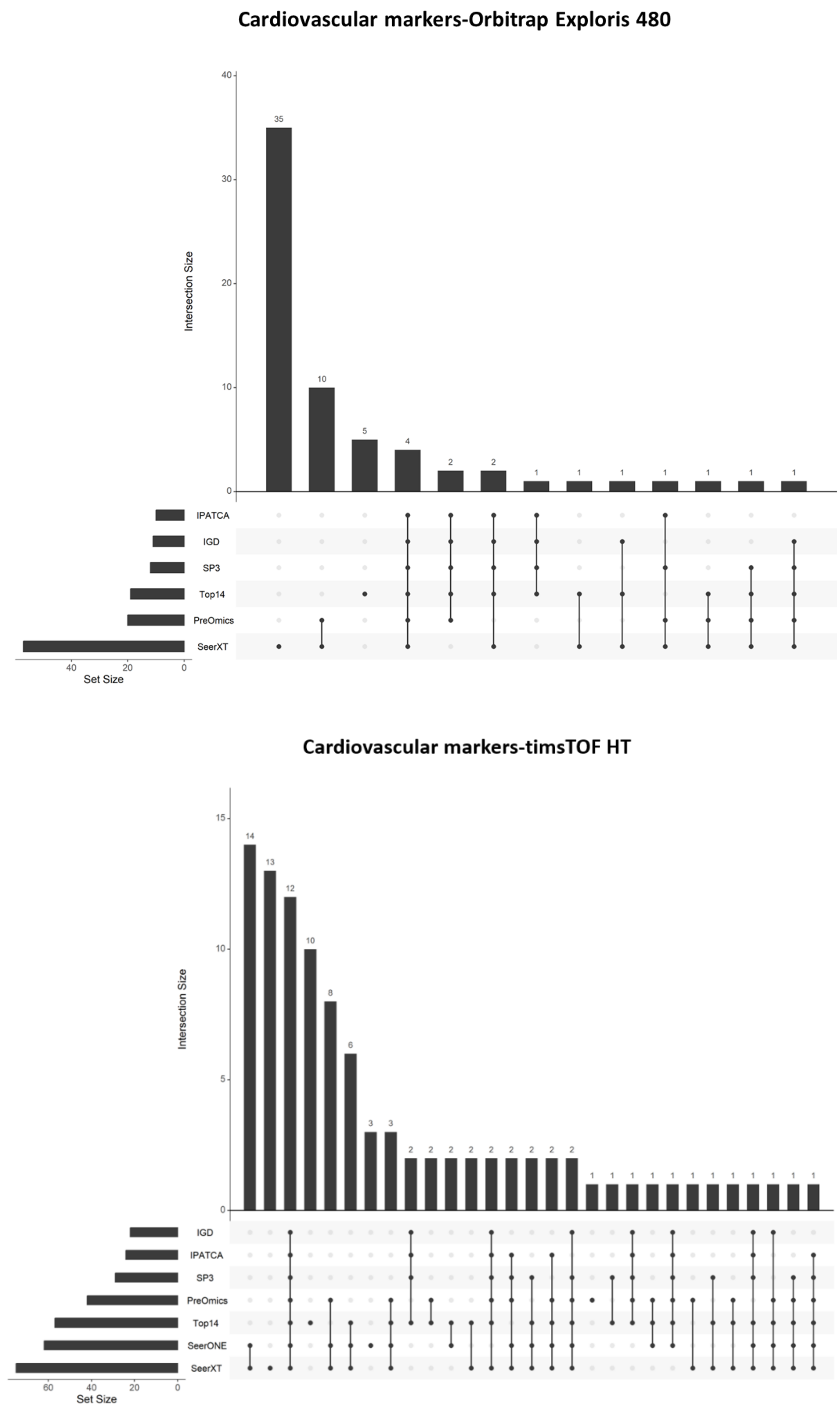

Supporting Figure S6. Overlaps of the disease marker panels across sample preparation methods on both MS platforms.

Supporting Tables

Supporting Table S1. Concentration ranges of the 7 spiked-in serum proteins

Gene Name	Protein description	Provider	Control	Spike 1 (ng/µl)	Spike 2 (ng/µl)	Spike 3 (ng/µl)	Spike 4 (ng/µl)
CRP	C-reactive protein	Sigma-Aldrich	-	4	2	1	0.5
S100A8	Protein S100-A8	ACROBiosystems	-	4	2	1	0.5
LYZ	Lysozyme C	Abcam	-	2	1	0.5	0.25
PPIA	Peptidyl-propyl cis-trans isomerase A	Abcam	-	2	1	0.5	0.25
YWHAE	14-3-3 protein epsilon	Abbexa	-	0.5	0.25	0.125	0.0625
GLO1	Lactoylglutathione lyase	Abbexa	-	0.4	0.2	0.1	0.05
PRKAR1A	cAMP-dependent protein kinase type I-alpha regulatory subunit	Abcam	-	0.4	0.2	0.1	0.05

Supporting Table S2. Average number of peptides per protein group across sample preparation methods and MS platforms

Sample preparation method	Orbitrap Exploris 480	timsTOF HT
**IGD**	10.16	10.3
**SP3**	11.59	10.75
**IPA/TCA**	9.97	9.3
**Top 14**	11.96	10.67
**PreOmics**	8.26	7.96
**Seer XT**	6.55	6.91
**Seer ONE**		8.08

Supporting Table S3. Median peptide yield per serum input across sample preparation methods

Sample preparation method	Starting material (µl)	Median peptide yield (µg)
**IGD**	1	18.85
**SP3**	1	26.9
**IPA/TCA**	10	123.22
**Top 14**	10	4.95
**PreOmics**	20	17.46
**Seer XT**	100 + 100	1.52
**Seer ONE**	120	1.17

Supporting Table S4. Peak capacity values across sample preparation methods and MS platforms

Sample preparation method	Orbitrap Exploris 480	timsTOF HT
**IGD**	276.18	280.51
**SP3**	243.28	297.99
**IPA/TCA**	188.81	285.17
**Top 14**	183.18	299.12
**PreOmics**	277.25	285.38
**Seer XT**	297.55	293.44
**Seer ONE**		279.14

Supporting Table S5. Quantitative reproducibility using average CV% across all replicates of spiked-in proteins (reported as HGNC gene symbols) for all workflows on timsTOF HT

Sample preparation method	High concentration		Medium concentration		Low concentration		
	**CRP**	**S100A8**	**LYZ**	**PPIA**	**YHWAE**	**GLO1**	**PRKAR1A**
**IGD**	4.35	8.81	9.13	5.84	18.58	22.45	18.66
**SP3**	8.15	4.44	12.54	13.83		18.71	14.65
**IPA/TCA**	7.00	14.92	8.85	8.47		11.38	11.12
**Top14**	4.69	6.65	4.03	9.12	9.22	12.91	13.37
**PreOmics**	12.36	8.32	5.82	7.12	23.36	5.60	8.47
**Seer XT**	11.23	8.55	10.85	4.90	5.15	7.94	5.57
**Seer ONE**	8.16	14.42	8.07	7.42	19.48	19.01	4.89

Supporting Table S6. Quantitative reproducibility using average CV% across all replicates of spiked-in proteins (reported as HGNC gene symbols) for all workflows on Orbitrap Exploris 480

Sample preparation method	High concentration		Medium concentration		Low concentration		
	CRP	S100A8	LYZ	PPIA	YWHAE	GLO1	PRKAR1A
IGD	9.15	13.64	16.18	11.48			
SP3	6.18	15.77	14.21	21.68			16.74
IPA/TCA	2.77	19.89	21.52	16.45			
Top 14	4.19	8.09	5.26	15.47	17.06		27.86
PreOmics	6.41	5.55	4.40	5.40		12.77	10.08
Seer XT	7.04	6.57	11.86	5.87	10.70	5.86	8.14

Supporting Table S7. Endogenous concentrations (ng/µl) of spiked-in proteins (reported as HGNC gene symbols) for each sample preparation method on timsTOF HT

Sample preparation method	High concentration		Medium concentration		Low concentration		
	CRP	S100A8	LYZ	PPIA	YHWAE	GLO1	PRKAR1A
IGD	1.912	0.816	0.879	1.412	0.240	0.009	0.039
SP3	2.304	3.435	1.346	0.621			0.020
IPA/TCA	1.872	0.001	0.745	0.574			
Top14	2.417	0.242	0.951	0.536	0.389		
PreOmics	2.193	0.177	0.930	0.027			0.020
Seer XT	0.579	0.276	0.889	0.992	0.064		0.118
Seer ONE	0.039	0.314	1.159	1.150	0.074		0.184

Supporting Table S8. Endogenous concentrations (ng/µl) of spiked-in proteins (reported as HGNC gene symbols) for each sample preparation method on Orbitrap Exploris 480

Sample preparation method	High concentration		Medium concentration		Low concentration		
	CRP	S100A8	LYZ	PPIA	YWHAE	GLO1	PRKAR1A
IGD	1.551	0.448	0.736	2.193			
SP3	2.457	1.290	0.855	1.622			
IPA/TCA	1.632		1.030	0.916			
Top 14	1.672	0.101	0.733	0.654			
PreOmics	2.107	0.128	0.721	0.028			
Seer XT	0.534	0.046	0.901	0.986	0.060		

Supporting Table S9. Number of identified stripped peptide IDs for spiked-in proteins (reported as HGNC gene symbols) across all methods on timsTOF HT

Sample preparation method	High concentration		Medium concentration		Low concentration		
	CRP	S100A8	LYZ	PPIA	YHWAE	GLO1	PRKAR1A
IGD	7	7	7	6	5	6	9
SP3	7	7	10	6	0	5	8
IPA/TCA	8	7	5	8	0	5	7
Top14	7	10	9	6	12	9	15
PreOmics	6	10	7	6	3	9	15
Seer XT	5	15	7	7	13	12	12
Seer ONE	5	12	15	7	10	11	14

Supporting Table S10. Number of identified stripped peptide IDs for spiked-in proteins (reported as HGNC gene symbols) across all methods on Orbitrap Exploris 480

Sample preparation method	High concentration		Medium concentration		Low concentration		
	CRP	S100A8	LYZ	PPIA	YWHAE	GLO1	PRKAR1A
IGD	6	6	5	6	0	0	0
SP3	5	5	7	7	0	0	3
IPA/TCA	6	2	2	5	0	0	0
Top 14	6	8	5	3	1	0	9
PreOmics	5	12	8	5	0	6	12
Seer XT	5	11	5	6	14	11	11

Supporting Table S11. Precursor-level median CV% across all methods on Orbitrap Exploris 480 and timsTOF HT

Precursor-level Median CV%		
Sample preparation method	timsTOF HT	Orbitrap 480
IGD	30.70	28.9
SP3	40.10	33.50
IPA/TCA	30.20	22.90
Top14	30.30	26
PreOmics	26.4	28.20
Seer XT	53.4	55.20
